# Comparing Web-Based Platforms for Promoting HIV Self-Testing and Pre-Exposure Prophylaxis Uptake in High-Risk Men Who Have Sex With Men: Protocol for a Longitudinal Cohort Study

**DOI:** 10.2196/20417

**Published:** 2020-10-19

**Authors:** Shea M Lemley, Jeffrey D Klausner, Sean D Young, Chrysovalantis Stafylis, Caroline Mulatya, Neal Oden, Haiyi Xie, Leslie Revoredo, Dikla Shmueli-Blumberg, Emily Hichborn, Erin McKelle, Landhing Moran, Petra Jacobs, Lisa A Marsch

**Affiliations:** 1 Geisel School of Medicine Dartmouth College Lebanon, NH United States; 2 David Geffen School of Medicine University of California, Los Angeles Los Angeles, CA United States; 3 Department of Emergency Medicine School of Medicine University of California, Irvine Irvine, CA United States; 4 Department of Informatics Bren School of Information and Computer Sciences University of California, Irvine Irvine, CA United States; 5 The Emmes Company, LLC Rockville, MD United States; 6 ETR Associates (Education, Training and Research) Oakland, CA United States; 7 National Institute on Drug Abuse Bethesda, MD United States

**Keywords:** HIV, HIV self-testing, men who have sex with men, web-based platforms, online advertising, social media, dating apps, informational search websites

## Abstract

**Background:**

The majority of those living with HIV in the United States are men who have sex with men (MSM), and young, minority MSM account for more new HIV infections than any other group. HIV transmission can be reduced through detection and early treatment initiation or by starting pre-exposure prophylaxis (PrEP), but rates of testing are lower than recommended among MSM, and PrEP uptake has been slow. Although promoting HIV testing and PrEP uptake by placing advertisements on web-based platforms — such as social media websites and dating apps — is a promising approach for promoting HIV testing and PrEP, the relative effectiveness of HIV prevention advertising on common web-based platforms is underexamined.

**Objective:**

This study aims to evaluate the relative effectiveness of advertisements placed on 3 types of web-based platforms (social media websites, dating apps, and informational websites) for promoting HIV self-testing and PrEP uptake.

**Methods:**

Advertisements will be placed on social media websites (Facebook, Instagram, and Twitter), dating apps (Grindr, Jack’d, and Hornet), and informational search websites (Google, Yahoo, and Bing) to recruit approximately 400 young (18-30 years old), minority (Black or Latino) MSM at elevated risk of HIV exposure. Recruitment will occur in 3 waves, with each wave running advertisements on 1 website from each type of platform. The number of participants per platform is not prespecified, and recruitment in each wave will occur until approximately 133 HIV self-tests are ordered. Participants will complete a baseline survey assessing risk behavior, substance use, psychological readiness to test, and attitudes and then receive an electronic code to order a free home-based HIV self-test kit. Two follow-ups are planned to assess HIV self-test results and PrEP uptake.

**Results:**

Recruitment was completed in July 2020.

**Conclusions:**

Findings may improve our understanding of how the platform users’ receptivity to test for HIV differs across web-based platforms and thus may assist in facilitating web-based HIV prevention campaigns.

**Trial Registration:**

ClinicalTrials.gov NCT04155502; https://clinicaltrials.gov/ct2/show/NCT04155502

**International Registered Report Identifier (IRRID):**

DERR1-10.2196/20417

## Introduction

Despite accounting for a small fraction of the US population (6%), men who have sex with men (MSM) account for the majority (61%) of those infected with human HIV in the United States and experience the largest burden (69%) of new infections [1-3]. More than 30% of new infections occur in young (under 35 years of age) MSM who identify as Black or Hispanic/Latino [3]. Most new HIV infections are the result of male-to-male sexual contact [2], but other behaviors elevate risk of HIV transmission. For example, substance use is prevalent among young MSM [4], and use of substances, particularly alcohol and amphetamines, is associated with not using a condom, a key risk factor for transmission of HIV infection [5-7].

Although minority MSM are not more likely to engage in risky sexual behavior than other MSM [8,9], Black and Hispanic/Latino MSM are disproportionately affected by societal factors — such as discrimination, lack of social support, and financial barriers — that are associated with problematic substance use and elevated HIV risk behavior [10,11]. Among minority MSM, experiences of racism are associated with greater likelihood of stimulant and polydrug use in the past 6 months [12]. Relative to other MSM, Black MSM have lower levels of social support that may drive higher rates of substance dependence and transactional sex [13], which further increase the risk of HIV transmission. In addition, some young Black and Latino MSM use social media websites and dating apps to exchange sex for money or drugs [14]. Cultural variables are also associated with elevated HIV risk behavior. For example, among Latino MSM, greater endorsement of cultural beliefs about gender and sexuality is related to HIV risk behaviors, such as unprotected anal intercourse [15,16]. Similarly, perceptions of imbalanced masculinity within partner dyads can create imbalanced partner dynamics among young Black MSM that negatively impact condom use [17].

HIV testing and treatment initiation can reduce the transmission of infection by 95% [18]. Approximately 1.1 million people in the United States are living with an HIV infection, and 14% (1 in 7) of those people are unaware they are infected [19]. Although the Centers for Disease Control and Prevention (CDC) recommends HIV testing at least every 6 months for those at elevated risk of HIV transmission [19], only 85% of internet-using MSM have been tested for HIV infection, and only 58% had been tested in the last year [20]. Younger MSM (<30 years old) are moderately more likely to never have been tested for HIV [19,20], and common barriers to HIV testing, such as not having insurance or transportation to a testing place, are reported more by younger MSM [21,22]. Substance use is also associated with decreased odds of recent HIV testing and increased likelihood of unknown HIV infection [23,24], suggesting an unmet need for HIV testing promotion, particularly among younger MSM and those who use substances.

HIV transmission rates can also be reduced with pre-exposure prophylaxis (PrEP). PrEP is the combination of 2 antiviral drugs in the form of a pill that, when taken, regularly prevents new infections. Randomized controlled trials in high-risk populations have shown that infection rates with PrEP use drop dramatically, to as low as 0% (eg, [25]). PrEP is only available by prescription, and the CDC recommends prescribing PrEP to all adults with substantial risk for HIV infection [26]. Nevertheless, PrEP awareness [27] and, most importantly, uptake have been slow, especially among young Black and Hispanic/Latino MSM [28,29]. Among young MSM, concerns about stigma from medical providers and concerns about paying for PrEP were significantly associated with reduced likelihood of PrEP use, even though PrEP assistance programs are available to help those who do not have health insurance that covers PrEP [30].

Using online approaches to promote HIV testing and PrEP uptake is a promising avenue for HIV prevention. Nearly 7 of 10 Americans use social media to connect with one another, engage with news content, share information, and entertain themselves [31]. Compared to all other age groups, regardless of sexual preference, those between 18 and 30 years of age comprise the group that is most likely to be active and engaged in daily social media use [32]. Among MSM, 97% use social media [33], and previous studies have used social media websites (eg, Facebook) for a range of HIV prevention efforts [34,35]. Young MSM also frequently use dating apps like Grindr to connect with new sex partners [32]. Researchers have also used dating apps to promote HIV prevention, such as by distributing HIV self-testing kits [36]. Social media websites and dating apps target health promotion advertising based on user demographics and behavior, while informational search websites (eg, Google, Bing, and Yahoo) focus on advertising to users by integrating data from what is privately typed into search bars. MSM may privately search for HIV prevention-related materials, even if they do not publicly post content about personal HIV prevention interests to social media websites. Trends in search data from informational search websites predict new diagnoses of sexually transmitted infections, including HIV, at the community level [37,38]. Although different in nature from social media websites, informational search websites also represent a highly promising additional avenue for outreach. Little is known, however, about the relative effectiveness of different web-based platforms (ie, social media websites, informational search websites, and dating apps) in promoting HIV testing and PrEP use. 

In addition, key factors that differentiate or moderate web-based platform users’ receptivity to HIV testing are less understood. For example, although substance use is associated with lower rates of HIV testing among MSM, how substance use impacts the likelihood of HIV self-testing when MSM are recruited online is underexamined. This study seeks to address those questions by evaluating the effectiveness of online campaigns promoting HIV self-testing on different types of web-based platforms: social media websites (Facebook, Instagram, Twitter), dating apps (Grindr, Jack’d, Hornet), and informational search websites (Google, Bing, Yahoo). Factors that moderate platform users’ testing receptivity across web-based platforms will also be examined.

The primary objective of this study is to compare HIV self-testing uptake among users of the 3 different types of web-based platforms. Secondary aims will seek to evaluate differences in PrEP uptake, as well as the impact of key moderator variables — problematic substance use and psychological readiness to test — on HIV testing and PrEP uptake. Other secondary aims include determining the efficiency of the different platform types for promoting HIV testing and PrEP uptake and evaluating the impact of perceptions and attitudes on HIV testing and PrEP uptake.

## Methods

### Study Design

This will be a longitudinal cohort study recruiting participants from 3 types of web-based platforms: social media websites, dating apps, and informational websites. The study team will develop a culturally appropriate, community-tested study advertisement for use on the social media websites and dating apps and will develop a list of keywords for advertising on informational search websites. The study advertisement will be placed on each platform, and similar campaigns will be employed across all 3 web-based platforms with the same money budgeted for advertising.

Recruitment will occur in 3 waves. In each wave, the advertisement will be placed on 1 social media website, 1 dating app, and 1 informational search website (see Table 1), for a total of 3 social media websites, dating apps, and informational search websites. To address the possibility of history effects occurring across waves, the study team selected a social media website, a dating app, and an informational search website to include in each wave. There will not be a prespecified number of participants recruited per platform/website (social media websites, informational search websites, or dating apps). Instead, recruitment in each wave will continue until approximately 133 self-test kits are ordered from across the 3 websites in that wave. Advertising in each wave will be scheduled to run for 30 calendar days. This duration was selected to optimize the advertisement placement given the advertising budget for each website. If at least 133 self-test kits are not ordered by the end of 30 calendar days, recruitment may be extended across all 3 websites to obtain the 133 self-test kit orders. 

**Table 1:** HIV self-test study recruitment waves and web-based platforms.

Recruitment wave	Informational search website	Social media website	Dating app
1	Google	Facebook	Grindr
2	Bing	Instagram	Jack’d
3	Yahoo	Twitter	Hornet

Upon clicking the study advertisement, users will land on the study informational page in Qualtrics 2020 software [39], where they will receive information about the study and undergo eligibility screening. An electronic study information sheet describing study procedures, participant rights, and potential risks and benefits will be provided to eligible users to review and download. The consent form will include information on what information the websites will be able to access and how the study team will ensure participants’ confidentiality, with identifiable information stored separately from responses. Participants will consent to participate by clicking an “Agree” button and proceed to identity verification. If users are ineligible or decline to participate in the study, they will be redirected to a website with information about HIV and sexually transmitted disease prevention, PrEP, and HIV testing locations.

After consenting, participants will be asked to login to their Facebook account within Qualtrics via single sign-on (SSO) to verify their identity and reduce duplicate participation. The information obtained by the study team from the SSO feature is described in the consent form, and private data are stored securely. The SSO requires participants to have a Facebook account and may prevent or deter some people from participating [40], but this requirement was deemed to be important for preventing duplicate participation and test kit orders, given that the study procedures are entirely remote. After logging into Facebook, participants will continue in Qualtrics to complete the baseline assessment. Once the baseline assessment has been completed, study staff will verify that the Facebook account is active (ie, has a photo and >10 friends) and that the participation is not a duplicate (ie, name, contact information, and Facebook account do not match any enrolled participants). If the participation is not a duplicate, site staff will email an electronic code for the participant to order 1 free HIV self-test kit. Duplicate participations will not receive a self-test kit code, gift card, or invitation to participate in follow-ups. Each eligible individual enrolled in the study can only order 1 HIV self-test kit. The study team will send approximately 400 coupon codes redeemable for free OraQuick Home HIV self-test kits [41] to eligible participants. The OraQuick Home HIV test kit is a Federal Drug Administration–approved self-test kit for home use that detects HIV antibodies in oral fluid in approximately 20 minutes. The kit is commercially available in pharmacies and online. The test kit includes a pretest counseling pamphlet and access to OraSure customer support by website and phone. In-home self-test kits have high acceptability among MSM [42], and prior research has found the OraQuick test kit to be easy to use [36].

We will follow up with each participant at 2 intervals, 14 days and 60 days after baseline, to evaluate our study objectives. Each participant will receive a US $25 electronic gift card upon completion of each evaluation (baseline, 14-day follow-up, and 60-day follow-up) for a maximum total of US $75.

### Study Population

The study aims to recruit approximately 400 MSM (or an appropriate number of participants until approximately 400 HIV self-test kits have been ordered) aged 18-30 years, inclusive, who are at increased risk of HIV exposure or infection and who use social media websites, dating apps, or informational search websites. Participants will have to self-report being Latino and/or Black/African American men (including multiracial and multiethnic members of these groups) and report high-risk sexual behavior with men (such as both condomless anal sex in the past 90 days and more than 1 sexual partner in the past 90 days). Participants must report not currently being on PrEP, not having taken PrEP in the past 6 months, and not having been tested for HIV in at least the past 3 months.

### Study Sites

#### Web-Based Platforms and Websites

Recruitment for this study will occur online through blast advertisements placed on 1 of 3 types of web-based platforms: social media websites, dating apps, and informational search websites. We selected 9 websites (3 of each platform type) that are popular among the study population (MSM aged 18-30 years old) and allow us to place location-targeted advertisements. Although some websites offer additional targeting options (eg, LGBTQ interests), no additional targeting was specified to keep conditions similar across platforms. During each wave, we will allocate approximately US $1100 weekly for advertising on each website.

For social media websites, we selected Facebook, Instagram, and Twitter. Facebook is a social networking website accessible through a dedicated smartphone app or a web browser. Of US adults aged 18-29 years, 81% own and use a Facebook account [32]. Facebook has been frequently used in many studies as a means for recruitment and prevention message dissemination [32,43]. Our team has previously used Facebook to recruit and retain MSM for 12 months [44,45]. Instagram is a photo-sharing social networking app that can be accessed by a smartphone app or web browser. As of June 2018, Instagram reported more than 1 billion monthly active users worldwide, mostly younger users below the age of 35 years [46]. In a recent study among focus groups of Black college students, participants indicated Instagram was one of their preferred social networks, underscoring the increased popularity and usage of these websites for young people (18-29 years of age) and non-Hispanic Black people [35]. Twitter is a news and social networking service accessible by web browser and a dedicated app. There are over 69 million Twitter users in the United States, and an average 36% of Americans aged 18-29 years use Twitter [47].

Grindr, Jack’d, and Hornet were selected as dating apps. Grindr, which is accessible only via its dedicated smartphone app, is the largest social networking app for gay, bisexual (“bi”), transgender (“trans”), and queer people [48]. Multiple studies have used Grindr as a platform to reach and recruit study participants (eg, [49,50]). Our team has used Grindr for advertising and has reached nearly 12,000 unique users, which resulted in 334 HIV self-test kit orders [49]. Jack’d is a location-based mobile app for gay and bisexual men to meet other men. The app has a global network of 1.2 million users. Nearly 80% of its users are under 24 years of age, while 30% of its users are Black, and 20% are multiracial or Latino [51]. Hornet is accessible via smartphone app and a web-based platform. It has nearly 3 million users in the United States, and its most active users are gay men aged 18-34 years. In the United States, 16% of Hornet users are Latinos, and 9% are African Americans [52].

Google, Bing, and Yahoo were selected as the informational search websites for this study. These internet search engines are accessible from personal computers or handheld devices. Google received approximately 92% of search visits in the United States as of 2019 [53,54]. Bing received 3.9% of search visits, and Yahoo received approximately 3% of search visits [53,54].

**Figure 1:**
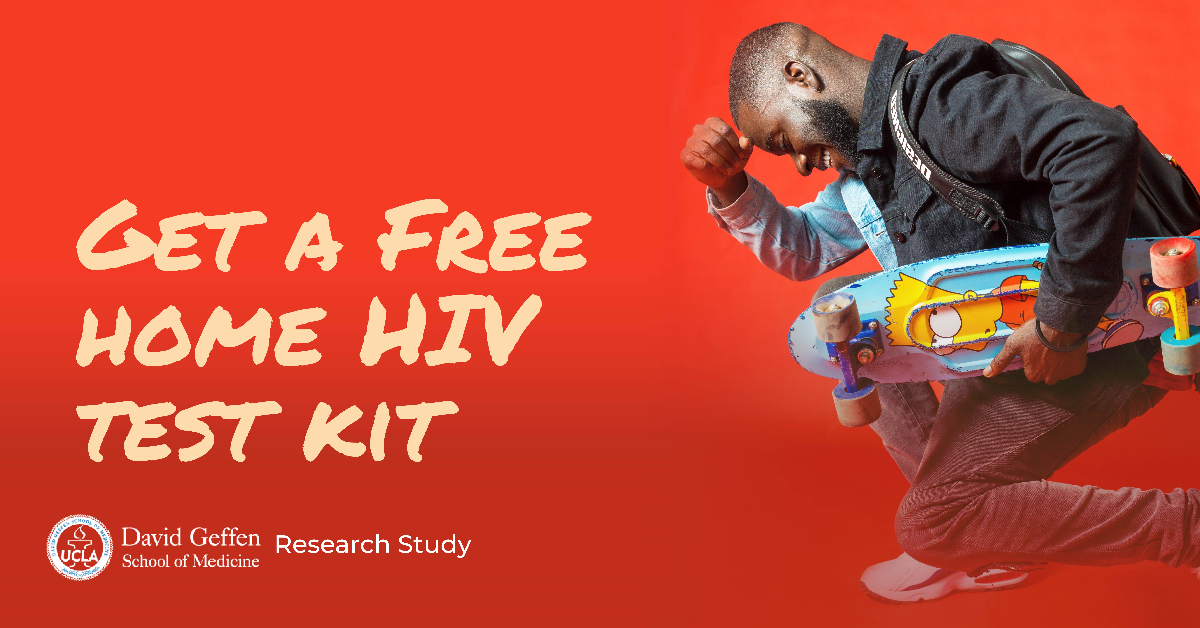
HIV self-test study advertisement developed by the study team for social media websites and dating apps.

The study team identified relevant topics and generated a list of keywords for the informational search websites, which were then tested with Google Trends to identify additional keywords and remove less relevant or unpopular words and phrases. Textbox 1 shows the final list of keywords used on the informational search websites. We developed potential advertisements, which were then modified through feedback with the study team and pilot testing. We pilot tested 2 advertisements using Facebook’s A/B testing, and Figure 1 shows the advertisement selected for use on the study platforms. The same image and text were used in all advertising placed on social media websites and dating apps.

**HIV self-test study keywords for informational search websites. Keywords and informational website searches were not case sensitive. Expansions of abbreviations shown in parentheses are included for clarity and were not included among the keywords.**

- HIV
- HIV symptoms
- Signs of HIV
- HIV AIDS
- Home HIV test
- Free HIV testing
- HIV positive
- How do you get HIV
- HIV test
- Free STD (sexually transmitted disease) testing
- Where to get HIV test
- PrEP (preexposure prophylaxis) HIV
- PrEP
- Preexposure prophylaxis
- Prevent HIV
- Prevent AIDS
- Truvada, Descovy
- AIDS HIV
- HIV and AIDS
- How do you get tested for HIV
- HIV negative
- Test for HIV
- HIV at home blood test
- At home early HIV test
- Over the counter at home HIV test kit
- AIDS and HIV
- Which HIV home test kit has FDA (Federal Drug Administration) approval
- Where to buy HIV home test kit
- Where to get HIV home test kit
- When to use HIV home test kit
- When to take HIV home test
- What is HIV home test kit
- HIV home test kit in stores
- Where to buy HIV test
- Where to do an HIV test
- Where to get an HIV test
- Where to get a free HIV test
- What is HIV home test
- Home test kit for HIV
- Home test kit for STD
- Where to get preexposure prophylaxis
- When to get preexposure prophylaxis
- When to get an HIV test
- HIV test without blood
- HIV test near me
- HIV test near me free
- HIV test nearby
- What HIV test can provide immediate results
- When HIV test can be done
- HIV test for free
- HIV test to buy
- HIV test to order
- HIV test at home
- HIV test with saliva
- HIV test with
- HIV test with swab
- FDA approved HIV home test
- Where to buy HIV home test kit
- Where to get HIV home test kit
- When to use HIV home test kit
- When to take HIV home test kit
- HIV home test near me
- HIV test home testing kits
- What is HIV home test kit
- Will PrEP prevent HIV

As part of systems testing, we ran a version of the advertisement that linked to Qualtrics on 6 of the 9 study websites (testing of the dating apps was precluded by the limited budget). That testing allowed us to revise our participant tracking system and selected website metrics. We also modified our informational search website keywords based on recommendations from the Google Ads Search Engine Optimization feature, which utilizes user search data to assist with keyword development.

#### Geographical Study Areas

We will place advertisements in the District of Columbia (D.C.) and 8 states: Florida, Georgia, Louisiana, Maryland, Mississippi, Nevada, South Carolina, and Texas. These areas were selected because they have high rates of new HIV infections (≥17 per 100,000 [2]), sufficient coverage for confirmatory testing (gettested.org [55]), and at least one organization that provides PrEP in the area (PrEPmenow [56]) to facilitate uptake of PrEP among study participants (see Multimedia Appendix 1 for details).

### Study Assessments and Data Collection

The study assessments and time points are shown in Table 2 (see Multimedia Appendix 2 for copy of study assessments). Participants will complete the self-administered baseline assessment online via Qualtrics after electronically consenting and logging into Facebook SSO. As part of baseline, they will provide demographic and contact information, and then participants will report sexual risk behaviors using a subset of questions from the Rapid HIV Behavioral Assessment [57] and social media activity [44]. Recent substance use will be reported using a subset of the Tobacco, Alcohol, Prescription medications, and other Substance use (TAPS) tool [58] that will assess past 3-month use of alcohol, cannabis, cocaine and amphetamines, heroin, prescription drugs, and other illicit substances (eg, 3,4-methylenedioxy-methamphetamine, psilocybin, alkyl nitrites, lysergic acid diethylamide, gamma-hydroxybutyrate, ketamine). Participants will also answer a single-item Transtheoretical Model of Health Behavior Change question assessing readiness to test for HIV [59], a 1-question item extracted from the HIV Testing Questions - CDC that evaluates reasons for not testing for HIV [60], and the Medical Mistrust Inventory [61]. Additional measures will assess attitudes toward HIV testing [62], attitudes toward HIV treatment [63], HIV-related stigma [64], and sexual delay discounting [65], which assesses the participant’s likelihood of waiting for access to a condom. At the 14-day follow-up, participants will be emailed a link to a Qualtrics survey. Participants will report their self-test kit use and optionally upload a picture of their test result in the survey. If a participant elects to upload a photo, the study team will confirm the self-reported result using the manufacturer’s instructions [21]. If participants report a negative test result, they will be asked if they visited a PrEP provider, if they started PrEP, their opinions about PrEP, and facilitators and barriers [66]. If participants report a preliminary positive on the HIV self-test, they will be asked whether they visited an HIV treatment provider. At the 60-day follow-up, all participants will be asked to respond to study evaluation questions. If participants reported not testing or not starting PrEP at the 14-day follow-up, they will be asked those questions again. Although not part of study data collection, additional outreach attempts will be conducted by email and phone for those with positive or indeterminate test results to encourage confirmatory testing and linkage to care.

**Table 2:** HIV self-test study schedule of study assessments.

Measure	Baseline	14–day follow-up	60–day follow-up
Social Media Activity [44]	X^a^		
Rapid HIV Behavioral Assessment [57]	X		
Tobacco, Alcohol, Prescription medications, and other Substance use (TAPS) Tool [58]	X		
The Transtheoretical Model of Health Behavior Change (State of Change): HIV Testing [59]	X		
Attitudes Toward HIV Testing [62]	X		
Reasons for Not Testing for HIV [60]	X		
Attitudes Toward HIV Treatment [63]	X		
HIV-Related Stigma [64]	X		
Medical Mistrust Inventory [61]	X		
Sexual Delay Discounting [65]	X		
HIV Self-Test Result		X	*^b^
PrEP^c^ Uptake Facilitators & Barriers [66]		X	*
PrEP Opinions		X	*
Study Evaluation Questions			X

^a^X: assessed.

^b^*: assessed if not reported at 14-day follow-up.

^c^PrEP: preexposure prophylaxis.

Additional data will be collected from websites and online services. Each recruitment website will provide advertising metrics (eg, cost, impressions, clicks), and the advertisement placed on each platform will link to a separate Qualtrics screening and baseline survey to track from which platform participants were recruited. Reports on HIV self-test kit orders will be obtained directly from OraSure, allowing objective measurement in addition to the self-report data collected during follow-ups.

### Outcome Measures

The primary outcome is the number of HIV self-test kits ordered per day through each type of online platform (social media websites, informational search websites, dating apps) during the time each wave is operational. This will be measured by the number of HIV self-test kits ordered through the OraSure website by the type of online platform. This outcome was specified as a rate because of the expectation that different waves may be open for recruitment for different periods. Secondary outcomes include the number of participants who started PrEP and the number of participants who tested positive for HIV. Secondary outcomes will also assess whether those with more complex substance use history and severity — as measured using the validated categories of the TAPS [58] — will be less likely to order an HIV self-test kit and less likely to uptake PrEP. Similarly, we will examine whether those closer to the “action” (ie, Determination) stage for HIV testing will be more likely to be recruited through informational sites and will be more likely to order an HIV home self-test kit and start PrEP [59]. Other measures will characterize the sample or be assessed as potential moderators of HIV testing and PrEP uptake outcomes: attitudes toward HIV testing and treatment, HIV-related stigma, medical mistrust, sexual delay discounting, and PrEP facilitators and barriers. The amount of money spent per test kit ordered per promotion type, including all the costs of the intervention (advertisement, test kit), will be compared to determine the most efficient web-based platform for advertisements related to promotion of HIV home self-test kits and PrEP uptake.

### Sample Size Justification

The relative effectiveness of the 3 platform types will be evaluated by comparing the rates of test kit orders. The study will recruit a sufficient number of MSM who actively use social media websites, dating apps, and informational search websites to obtain approximately 400 test kit orders. The sample size justification involved investigating power for selected data aspects and estimating platform rates and their confidence intervals by specifying different effect sizes. The simulations assumed that each wave would be open for recruitment until at least 133 test kits were ordered across all 3 websites in that wave. The time each wave was open for enrollment was used as an offset in the Poisson regression model implemented in the simulations. The covariates in this Poisson model included wave, type of platform, and the interaction between the two. Power was investigated for data aspects such as for the test of the null hypothesis that the rates for the 3 dating apps can be pooled and for the test of the null hypothesis that the pooled rates (ie, across waves) of social media websites and dating apps are the same. Regarding the estimation of the rate for the dating app Grindr, the results showed that if the true Grindr rate is 40 kits per 2 weeks, the majority (at least 80%) of the 95% CIs for the Grindr rate will lie within 31 and 62 kits. If the true Grindr value is 10 kits per 2 weeks, the 95% CI will probably lie between 4 and 19. For the test of whether the rates of the 3 dating app websites can be pooled, we expect at least 80% power if the range of the rates exceeds 12 kits per 2 weeks. For the test of whether the pooled rates of social media websites and dating apps are the same, there is at least 80% power if the absolute difference is 8 kits per 2 weeks.

### Statistical Analysis

The platforms and their implementation are provided in Table 1. There are 3 rows corresponding to the 3 waves and 3 columns corresponding to the 3 types of promotional platforms. For each wave, the number of kits ordered from each website will be expressed as a daily rate (ie, number of kits ordered in that website divided by days to recruit 133 test kit orders). All kits ordered while the wave is actively recruiting will be included in the primary outcome analysis. It is hypothesized that the rate of kit orders will vary across platforms. The primary outcome analysis will be conducted using a Poisson regression model with wave recruitment time as an offset. The model will adjust for wave, type of website, and their interaction. A similar model will be used for the PrEP uptake secondary outcome. The additional secondary and exploratory analyses will be conducted using univariate tests and descriptive statistics such as counts, percentages, and 95% CIs.

## Results

The protocol and informed consent document were reviewed and approved by the University of California, Los Angeles Institutional Review Board. We started recruitment in January 2020, and recruitment was expected to take up to 9 months. Recruitment was completed in July 2020. Follow-up data collection was completed in September 2020.

## Discussion

This study seeks to evaluate the effectiveness of an online campaign promoting HIV self-testing on different types of web-based platforms: social media websites, dating apps, and informational search websites. The platforms’ relative effectiveness for promoting HIV self-testing will be determined by their rates of HIV test kit orders. The order rate for each platform may reflect the number of users, the number of high-risk users, and whether the users of that platform are likely to order a kit. For example, targeted analyses may demonstrate differences between the platforms in terms of their ability to reach a larger number of eligible participants quickly, as measured by a higher click volume, versus their ability to appeal to individuals that are more likely to ultimately order a test kit, as measured by a higher proportion of successful clicks. Secondary outcomes will include the number of participants who started PrEP, the number who tested positive for HIV, how these key outcomes are impacted by variables such as substance use, and the efficiency of the online platforms types for promoting HIV testing and PrEP uptake.

This study has implications for future research and public health promotion. Although previous studies have used dating and social media websites to promote HIV testing, to our knowledge, this is the first study to include informational search websites in an evaluation of online HIV prevention efforts. Findings may contribute to our understanding of the receptivity of users from these different types of platforms to obtain HIV prevention services. Further, public health funds are limited, so it is important to study the relative costs of different approaches for promoting health behavior, including HIV testing and PrEP uptake. Another piece of critical information to understand is the drivers and moderators of online users’ HIV testing and PrEP uptake. For example, results may inform how substance use affects HIV self-testing when MSM are recruited online. Understanding these and other factors impacting an individual’s receptiveness to test could assist in maximizing the impact of prevention campaigns through these popular online platforms.

## Appendix

Multimedia Appendix 1 Table showing criteria for selection of geographical study areas.

Multimedia Appendix 2 Human Immunodeficiency Virus self-test study assessments.

## Abbreviations

CDC: Centers for Disease Control and Prevention
FDA: Federal Drug Administration
MSM: men who have sex with men
NIDA: National Institute on Drug Abuse
PrEP: pre-exposure prophylaxis
SSO: single sign-on
STD: sexually transmitted disease
TAPS: Tobacco, Alcohol, Prescription medications, and other Substance use

## References

[1] Rosenberg ES, Grey JA, Sanchez TH, Sullivan PS (2016). Rates of Prevalent HIV Infection, Prevalent Diagnoses, and New Diagnoses Among Men Who Have Sex With Men in US States, Metropolitan Statistical Areas, and Counties, 2012-2013. JMIR Public Health Surveill.

[2] Centers for Disease Control and Prevention (2018). HIV Surveillance Report: Diagnoses of HIV Infection in the United States and Dependent Areas, 2018 (updated).

[3] Centers for Disease Control and Prevention (2019). NCHHSTP AtlasPlus.

[4] Lelutiu-Weinberger C, Pachankis JE, Golub SA, Walker JJ, Bamonte AJ, Parsons JT (2013). Age cohort differences in the effects of gay-related stigma, anxiety and identification with the gay community on sexual risk and substance use. AIDS Behav.

[5] Lacefield K, Negy C, Schrader RM, Kuhlman C (2015). Comparing Psychosocial Correlates of Condomless Anal Sex in HIV-Diagnosed and HIV-Nondiagnosed Men Who Have Sex with Men: A Series of Meta-Analyses of Studies from 1993-2013. LGBT Health.

[6] Koblin BA, Murrill C, Camacho M, Xu G, Liu K, Raj-Singh S, Torian L (2007). Amphetamine use and sexual risk among men who have sex with men: results from the National HIV Behavioral Surveillance study--New York City. Subst Use Misuse.

[7] Bourne A, Weatherburn P (2017). Substance use among men who have sex with men: patterns, motivations, impacts and intervention development need. Sex Transm Infect.

[8] Holloway IW, Pulsipher CA, Gibbs J, Barman-Adhikari A, Rice E (2015). Network Influences on the Sexual Risk Behaviors of Gay, Bisexual and Other Men Who Have Sex with Men Using Geosocial Networking Applications. AIDS Behav.

[9] Millett GA, Peterson JL, Wolitski RJ, Stall R (2006). Greater risk for HIV infection of black men who have sex with men: a critical literature review. Am J Public Health.

[10] Ayala G, Bingham T, Kim J, Wheeler DP, Millett GA (2012). Modeling the Impact of Social Discrimination and Financial Hardship on the Sexual Risk of HIV Among Latino and Black Men Who Have Sex With Men. Am J Public Health.

[11] Buttram ME, Kurtz SP (2015). A mixed methods study of health and social disparities among substance-using African American/Black men who have sex with men. J Racial Ethn Health Disparities.

[12] Paul JP, Boylan R, Gregorich S, Ayala G, Choi K (2014). Substance use and experienced stigmatization among ethnic minority men who have sex with men in the United States. J Ethn Subst Abuse.

[13] Buttram ME, Kurtz SP, Surratt HL (2013). Substance use and sexual risk mediated by social support among Black men. J Community Health.

[14] Patel VV, Masyukova M, Sutton D, Horvath KJ (2016). Social Media Use and HIV-Related Risk Behaviors in Young Black and Latino Gay and Bi Men and Transgender Individuals in New York City: Implications for Online Interventions. J Urban Health.

[15] Lo SC, Reisen CA, Poppen PJ, Bianchi FT, Zea MC (2011). Cultural beliefs, partner characteristics, communication, and sexual risk among Latino MSM. AIDS Behav.

[16] Jarama SL, Kennamer JD, Poppen PJ, Hendricks M, Bradford J (2005). Psychosocial, behavioral, and cultural predictors of sexual risk for HIV infection among Latino men who have sex with men. AIDS Behav.

[17] Fields EL, Bogart LM, Smith KC, Malebranche DJ, Ellen J, Schuster MA (2012). HIV risk and perceptions of masculinity among young black men who have sex with men. J Adolesc Health.

[18] Cohen MS, Chen YQ, McCauley M, Gamble T, Hosseinipour MC, Kumarasamy N, Hakim JG, Kumwenda J, Grinsztejn B, Pilotto JHS, Godbole SV, Chariyalertsak S, Santos BR, Mayer KH, Hoffman IF, Eshleman SH, Piwowar-Manning E, Cottle L, Zhang XC, Makhema J, Mills LA, Panchia R, Faesen S, Eron J, Gallant J, Havlir D, Swindells S, Elharrar V, Burns D, Taha TE, Nielsen-Saines K, Celentano DD, Essex M, Hudelson SE, Redd AD, Fleming TR, HPTN 052 Study Team (2016). Antiretroviral Therapy for the Prevention of HIV-1 Transmission. N Engl J Med.

[19] US Department of Health and Human Services Overview: Data & Trends: U.S. Statistics. HIV.gov.

[20] Noble M, Jones AM, Bowles K, DiNenno EA, Tregear SJ (2017). HIV Testing Among Internet-Using MSM in the United States: Systematic Review. AIDS Behav.

[21] Margolis AD, Joseph H, Belcher L, Hirshfield S, Chiasson MA (2012). 'Never testing for HIV' among men who have sex with men recruited from a sexual networking website, United States. AIDS Behav.

[22] Mackellar DA, Hou S, Whalen CC, Samuelsen K, Sanchez T, Smith A, Denson D, Lansky A, Sullivan P, WHBS Study Group (2011). Reasons for not HIV testing, testing intentions, and potential use of an over-the-counter rapid HIV test in an internet sample of men who have sex with men who have never tested for HIV. Sex Transm Dis.

[23] Carrico AW, Storholm ED, Flentje A, Arnold EA, Pollack LM, Neilands TB, Rebchook GM, Peterson JL, Eke A, Johnson W, Kegeles SM (2017). Spirituality/religiosity, substance use, and HIV testing among young black men who have sex with men. Drug Alcohol Depend.

[24] Morgan E, Khanna AS, Skaathun B, Michaels S, Young L, Duvoisin R, Chang M, Voisin D, Cornwell B, Coombs RW, Friedman SR, Schneider JA (2016). Marijuana Use Among Young Black Men Who Have Sex With Men and the HIV Care Continuum: Findings From the uConnect Cohort. Subst Use Misuse.

[25] Volk JE, Marcus JL, Phengrasamy T, Blechinger D, Nguyen DP, Follansbee S, Hare CB (2015). No New HIV Infections With Increasing Use of HIV Preexposure Prophylaxis in a Clinical Practice Setting. Clin Infect Dis.

[26] Centers for Disease Control and Prevention (2017). Preexposure Prophylaxis for the Prevention of HIV Infection in the United States – 2017 Update Clinical Practice Guideline.

[27] Eaton LA, Driffin DD, Bauermeister J, Smith H, Conway-Washington C (2015). Minimal Awareness and Stalled Uptake of Pre-Exposure Prophylaxis (PrEP) Among at Risk, HIV-Negative, Black Men Who Have Sex with Men. AIDS Patient Care STDS.

[28] Huang YA, Zhu W, Smith DK, Harris N, Hoover KW (2018). HIV Preexposure Prophylaxis, by Race and Ethnicity - United States, 2014-2016. MMWR Morb Mortal Wkly Rep.

[29] Snowden J, Chen Y, McFarland W, Raymond H (2017). Prevalence and characteristics of users of pre-exposure prophylaxis (PrEP) among men who have sex with men, San Francisco, 2014 in a cross-sectional survey: implications for disparities. Sex Transm Infect.

[30] Jaiswal J, Griffin M, Singer SN, Greene RE, Acosta ILZ, Kaudeyr SK, Kapadia F, Halkitis PN (2018). Structural Barriers to Pre-exposure Prophylaxis Use Among Young Sexual Minority Men: The P18 Cohort Study. Curr HIV Res.

[31] Pew Research Center (2019). Social media fact sheet: Demographics of social media users and adoption in the United States.

[32] Smith A, Anderson M (2018). Social media use in 2018. Pew Research Center.

[33] Community Marketing & Insights (2019). 13th Annual LGBTQ Community Survey USA Summary Report.

[34] Rhodes SD, McCoy TP, Tanner AE, Stowers J, Bachmann LH, Nguyen AL, Ross MW (2016). Using Social Media to Increase HIV Testing Among Gay and Bisexual Men, Other Men Who Have Sex With Men, and Transgender Persons: Outcomes From a Randomized Community Trial. Clin Infect Dis.

[35] Jones J, Carter B, Wilkerson R, Kramer C (2019). Attitudes toward HIV testing, awareness of HIV campaigns, and using social networking sites to deliver HIV testing messages in the age of social media: a qualitative study of young black men. Health Educ Res.

[36] Huang E, Marlin RW, Young SD, Medline A, Klausner JD (2016). Using Grindr, a Smartphone Social-Networking Application, to Increase HIV Self-Testing Among Black and Latino Men Who Have Sex With Men in Los Angeles, 2014. AIDS Educ Prev.

[37] Young SD, Torrone EA, Urata J, Aral SO (2018). Using Search Engine Data as a Tool to Predict Syphilis. Epidemiology.

[38] Young SD, Zhang Q (2018). Using search engine big data for predicting new HIV diagnoses. PLoS One.

[39] Version March 2020. Qualtrics.

[40] Bauer L, Bravo-Lillo C, Fragkaki E, Melicher W (2013). A Comparison of Users' Perceptions of and Willingness to Use Google, Facebook, and Google+ Single-Sign-On Functionality.

[41] OraQuick: In-Home HIV Test.

[42] Raymond HF, Bingham T, McFarland W (2008). Locating unrecognized HIV infections among men who have sex with men: San Francisco and Los Angeles. AIDS Educ Prev.

[43] Pedersen ER, Kurz J (2016). Using Facebook for Health-related Research Study Recruitment and Program Delivery. Curr Opin Psychol.

[44] Young SD, Cumberland WG, Nianogo R, Menacho LA, Galea JT, Coates T (2015). The HOPE social media intervention for global HIV prevention in Peru: a cluster randomised controlled trial. Lancet HIV.

[45] Young SD (2014). Social media technologies for HIV prevention study retention among minority men who have sex with men (MSM). AIDS Behav.

[46] Clement J (2020). Instagram - Statistics & Facts. Statista.

[47] Omnicore (2020). Twitter by the Numbers: Stats, Demographics & Fun Facts.

[48] (2020). About us. Grindr.

[49] Rosengren AL, Huang E, Daniels J, Young SD, Marlin RW, Klausner JD (2016). Feasibility of using Grindr to distribute HIV self-test kits to men who have sex with men in Los Angeles, California. Sex Health.

[50] Gibbs JJ, Rice E (2016). The Social Context of Depression Symptomology in Sexual Minority Male Youth: Determinants of Depression in a Sample of Grindr Users. J Homosex.

[51] (2020). Contact us: Advertise. Jack'd.

[52] (2020). Hornet Review. DatingScout.

[53] Statista (2020). Distribution of total and mobile organic search visits in the United States as of 1st quarter 2020, by engine.

[54] Merkle (2020). Digital marketing report.

[55] Find a PrEP provider. PleasePrEPme.org.

[56] Centers for Disease Control and Prevention (2020). GetTested: National HIV, STD, and Heptatitis Testing.

[57] Gallagher KM, Denning PD, Allen DR, Nakashima AK, Sullivan PS (2007). Use of rapid behavioral assessments to determine the prevalence of HIV risk behaviors in high-risk populations. Public Health Rep.

[58] McNeely J, Wu L, Subramaniam G, Sharma G, Cathers LA, Svikis D, Sleiter L, Russell L, Nordeck C, Sharma A, O'Grady KE, Bouk LB, Cushing C, King J, Wahle A, Schwartz RP (2016). Performance of the Tobacco, Alcohol, Prescription Medication, and Other Substance Use (TAPS) Tool for Substance Use Screening in Primary Care Patients. Ann Intern Med.

[59] Prochaska JO, Redding CA, Harlow LL, Rossi JS, Velicer WF (1994). The transtheoretical model of change and HIV prevention: a review. Health Educ Q.

[60] U.S. Centers for Disease Control HIV/STD Behavioral Surveillance Working Group (2001). Core Measure for HIV/STD Risk Behavior and Prevention: Questionnaire-Based Measurement for Surveys and Other Data Systems. UCLA Center for HIV Identification, Prevention and Treatment Services.

[61] LaVeist T, Isaac L, Williams K (2009). Mistrust of health care organizations is associated with underutilization of health services. Health Serv Res.

[62] Kalichman SC, Simbayi LC (2003). HIV testing attitudes, AIDS stigma, and voluntary HIV counselling and testing in a black township in Cape Town, South Africa. Sex Transm Infect.

[63] Stolte IG, Dukers NH, Geskus RB, Coutinho RA, de Wit JBF (2004). Homosexual men change to risky sex when perceiving less threat of HIV/AIDS since availability of highly active antiretroviral therapy: a longitudinal study. AIDS.

[64] EKOS Research Associates Inc (2012). 2012 HIV/AIDS Attitudinal Tracking Survey: Final Report. Public Health Agency of Canada.

[65] Johnson MW, Bruner NR (2012). The Sexual Discounting Task: HIV risk behavior and the discounting of delayed sexual rewards in cocaine dependence. Drug Alcohol Depend.

[66] Golub SA, Gamarel KE, Rendina HJ, Surace A, Lelutiu-Weinberger CL (2013). From efficacy to effectiveness: facilitators and barriers to PrEP acceptability and motivations for adherence among MSM and transgender women in New York City. AIDS Patient Care STDS.

