# Supplementary material for: Comparing Web-Based Platforms for Promoting HIV Self-Testing and Pre-Exposure Prophylaxis Uptake in High-Risk Men Who Have Sex With Men: Protocol for a Longitudinal Cohort Study
Source: JMIR Res Protoc. 2020 Oct 19;9(10):e20417. doi: 10.2196/20417 (PMC7605984; doi:10.2196/20417)
Supplement: Multimedia Appendix 1 [file resprot_v9i10e20417_app1.docx]

**Supplemental Table: Site Selection Characteristics**

| **State** | **Diagnoses of HIV in adults and adolescents (2016 & 2017) [2]** | | **Confirmatory test referral sites [46]** | **PrEP support services [45]** | **Medicare/**  **Medicaid Expansion** | **Identified as Study Site** |
| --- | --- | --- | --- | --- | --- | --- |
|  | *Number* | *Rate*  *(per 100,000)* |  |  |  |  |
| Alabama | 665 | 16.3 | Yes | Yes | No |  |
| Alaska | 37 | 6.1 | Yes | Yes | Yes |  |
| Arizona | 722 | 12.6 | Yes | Yes | Yes |  |
| Arkansas | 310 | 12.5 | Yes | Yes | Yes |  |
| California | 5116 | 15.6 | Yes | Yes | Yes |  |
| Colorado | 424 | 9.2 | Yes | Yes | Yes |  |
| Connecticut | 261 | 8.5 | Yes | Yes | Yes |  |
| Delaware | 112 | 13.9 | Yes | Yes | Yes |  |
| DC | 343 | 58.2 | Yes | Yes | Yes | x |
| Florida | 4708 | 26.6 | Yes | Yes | No | x |
| Georgia | 2480 | 29.1 | Yes | Yes | No | x |
| Hawaii | 78 | 6.5 | Yes | Yes | Yes |  |
| Idaho | 47 | 3.4 | Yes | Yes | Yes |  |
| Illinois | 1472 | 13.7 | Yes | Yes | Yes |  |
| Indiana | 481 | 8.7 | Yes | Yes | Yes |  |
| Iowa | 133 | 5.1 | Yes | Yes | Yes |  |
| Kansas | 146 | 6.1 |  |  |  |  |
| Kentucky | 334 | 9.0 | Yes | Yes | Yes |  |
| Louisiana | 1122 | 28.9 | Yes | Yes | Yes | x |
| Maine | 53 | 4.6 | Yes | Yes | Yes |  |
| Maryland | 1104 | 21.8 | Yes | Yes | Yes | x |
| Massachusetts | 633 | 10.8 | Yes | Yes | Yes |  |
| Michigan | 745 | 8.9 | Yes | Yes | Yes |  |
| Minnesota | 292 | 6.4 | Yes | Yes | Yes |  |
| Mississippi | 429 | 17.4 | Yes | Yes | No | x |
| Missouri | 509 | 10.0 | Yes | Yes | No |  |
| Montana | 21 | 2.4 | Yes | Yes | Yes |  |
| Nebraska | 76 | 4.9 | Yes | Yes | Adopted |  |
| Nevada | 512 | 20.9 | Yes | Yes | Yes | x |
| New Hampshire | 40 | 3.5 | Yes | Yes | Yes |  |
| New Jersey | 1161 | 15.3 | Yes | Yes | Yes |  |
| New Mexico | 138 | 8.0 | Yes | Yes | Yes |  |
| New York | 2832 | 16.8 | Yes | Yes | Yes |  |
| North Carolina | 1392 | 16.3 | Yes | Yes | No |  |
| North Dakota | 45 | 7.2 | Yes | Yes | Yes |  |
| Ohio | 976 | 10.0 | Yes | Yes | Yes |  |
| Oklahoma | 293 | 9.1 | Yes | Yes | No |  |
| Oregon | 223 | 6.4 | Yes | Yes | Yes |  |
| Pennsylvania | 1138 | 10.4 | Yes | Yes | Yes |  |
| Rhode Island | 69 | 7.6 | Yes | Yes | Yes |  |
| South Carolina | 744 | 17.8 | Yes | Yes | No | x |
| South Dakota | 40 | 5.7 | Yes | Yes | No |  |
| Tennessee | 708 | 12.7 | Yes | Yes | No |  |
| Texas | 4508 | 19.9 | Yes | Yes | No | x |
| Utah | 137 | 5.8 | Yes | Yes | Yes |  |
| Vermont | 5 | 0.9 | Yes | Yes | Yes |  |
| Virginia | 902 | 12.8 | Yes | Yes | Yes |  |
| Washington | 428 | 7.0 | Yes | Yes | Yes |  |
| West Virginia | 67 | 4.3 | Yes | Yes | Yes |  |
| Wisconsin | 227 | 4.7 | Yes | Yes | No |  |
| Wyoming | 21 | 4.3 | Yes | Yes | No |  |
