# Supplementary material for: Comparing Web-Based Platforms for Promoting HIV Self-Testing and Pre-Exposure Prophylaxis Uptake in High-Risk Men Who Have Sex With Men: Protocol for a Longitudinal Cohort Study
Source: JMIR Res Protoc. 2020 Oct 19;9(10):e20417. doi: 10.2196/20417 (PMC7605984; doi:10.2196/20417)
Supplement: Multimedia Appendix 2 [file resprot_v9i10e20417_app2.docx]

Comparing Web-Based Platforms for Promoting HIV Self-Testing and PrEP Uptake in High-Risk MSM: Protocol for a Longitudinal Cohort Study

Study Assessments

Screening

Start of Block: Welcome Message

Q1.1

**Thank you for your interest in the study!!!** We are recruiting participants from different sites and apps. We are trying to find out which website(s) or app(s) are best for linking people to HIV self-testing and prevention information. Your responses could help us design prevention programs.

 **What happens if I join?** Answer a few screening questions to find out if you qualify Participate in a 20-minute survey Order a kit and test for HIV at home Get a gift card! Answer two more surveys. Each time you participate you get a gift card. If you are interested, answer the screening questions. We will ask you a few questions, such as your sex, age, sexual orientation. This will take up to 5 minutes. 
 Your participation in the screening is **voluntary**. You may leave this page any time.

 All your responses are **confidential.**
If you are eligible to participate, you will join the study, fill out another survey. We will give you more detailed information.

 If you are not eligible, we will keep your anonymous responses to understand how well our project did.
   For more information or if you have questions:   
• E-mail us at selftestnow@mednet.ucla.edu or call (310) 825-4321 
  • UCLA Office of the Human Research Protection Program (OHRPP): If you have questions about your rights as a research subject, or you have concerns or suggestions and you want to talk to someone other than the researchers, you may contact the UCLA OHRPP by phone: (310) 206-2040; by email: participants@research.ucla.edu or by mail: Box 951406, Los Angeles, CA 90095-1406   To learn more about HIV, please visit our study website [freehometest.org](http://freehometest.org/)   **Click the arrow to start!**

End of Block: Welcome Message

Start of Block: Age (Screening)

|  |
| --- |

Q3.1 How old are you?

________________________________________________________________

End of Block: Age (Screening)

Start of Block: Screening

Q4.1 What sex were you assigned at birth, on your original birth certificate?

- Male
- Female

| Page Break |  |
| --- | --- |

Q5.1 Are you Hispanic and/or Latino?

- Yes
- No

| Page Break |  |
| --- | --- |

Q5.2 What group represents your Hispanic origin or ancestry?

- Puerto Rican
- Dominican (Republic)
- Mexican/ Mexican American
- Chicano
- Cuban/ Cuban American
- Other ________________________________________________

| Page Break |  |
| --- | --- |

Q5.3
Do you self-identify as....

- American Indian or Alaska Native
- Asian
- Black or African American
- Native Hawaiian or Pacific Islander
- White
- Other, Please Specify ________________________________________________

| Page Break |  |
| --- | --- |

Q6.1 What is your HIV status?

- Negative
- Positive
- I don't know
- Refuse to answer

| Page Break |  |
| --- | --- |

Q6.3 In the past 90 days, did you have sex with:

- Men
- Women
- Transgender Men
- Transgender Women
- I didn't have sex in the past 90 days

| Page Break |  |
| --- | --- |

Q6.4 Have you tested for HIV in the past 90 days?

- Yes
- No

| Page Break |  |
| --- | --- |

Q6.2 Have you ever taken PrEP? (Also known as Pre-Exposure Prophylaxis, such as Truvada)

- Yes, but I haven't taken PrEP in the past six months
- Yes, I am currently taking PrEP
- No, I have never taken PrEP

| Page Break |  |
| --- | --- |

Q6.5 In the past 90 days, did you have anal sex without using a condom?

- Yes
- No

| Page Break |  |
| --- | --- |

Q6.6 In the past 90 days, did you have more than one male sex partner?

- Yes
- No

| Page Break |  |
| --- | --- |

Q6.7 Do you have a Facebook account? This is necessary to participate in the study.

- Yes
- No

| Page Break |  |
| --- | --- |

Q6.8
If you participate in the study, we will ask that you give us your email and your phone number.

We will use this information to contact you during your participation in this study, for example to send you links for the follow up surveys or send you the gift card.
 All information will be kept confidential. We will ask for this information later in the study. Are you okay with this?

- Yes
- No

End of Block: Screening

Consent

Start of Block: Eligible for the study

Q7.1
 Congratulations! You're eligible to join the study!! Before you can enroll in this study, please read the information below:   

 Download a copy (This will open a new window. You may return here at any point)
 
 
Do you agree to join the study?

- Yes
- No

Q7.2 Click below to proceed...

| Page Break |  |
| --- | --- |

End of Block: Eligible for the study

Baseline

Start of Block: Intro Baseline

Q8.1 Welcome to the study!
  Please answer the following questions as truthfully as possible.   Remember your answers are confidential.   We will email you instructions on how to order your test kit within 48 hours after you complete this questionnaire.
  
 Click the button below to start

Facebook Single Sign-on [not shown]

End of Block: Intro Baseline

Start of Block: Demographics

Q9.1

What is your relationship status?

- Single
- Married
- Widowed
- Divorced
- Separated
- In a relationship
- Other ________________________________________________
- Refuse to answer

| Page Break |  |
| --- | --- |

Q9.2 How much did you earn or receive from a job in the past MONTH?

- $0 - $2000
- $2001 - $5000
- $5001 or more
- Refuse to answer

| Page Break |  |
| --- | --- |

Q9.3 What social media sites do you use? Please check all that apply

- Facebook
- Tumblr
- Twitter
- Instagram
- Snapchat
- Grindr
- Hornet
- Jack'd
- Other, please specify ________________________________________________

| Page Break |  |
| --- | --- |

Q9.4 In the past three months, how much time did you spend on social media each day?

- I don't use social media
- 1 minute up to 2 hours
- 2-4 hours
- 4-6 hours
- 6-8 hours
- 8+ hours

| Page Break |  |
| --- | --- |

Q9.5 Why do you typically use social media? Check all that apply

- Research/general knowledge (looking up answers to questions)
- School/work
- News
- Messaging or email
- Finding sexual partners
- Entertainment (Music,videos, tv, chatting with friends)
- I don't use social media
- Other, please let us know ________________________________________________

| Page Break |  |
| --- | --- |

End of Block: Demographics

Start of Block: Contact information

Q10.1
Write down your email address and phone number.
 
We will use the email address to ***send you the information about the study, your gift card and instructions how to order a test kit***. We will also use this to contact you for the next follow-ups at 14 days and 60 days after today.
 
Please make sure that ***the information is accurate***.

- Email ________________________________________________
- Phone number (XXX-XXX-XXXX) ________________________________________________

End of Block: Contact information

Start of Block: Sexual Behavior & Risk Behavior

Q11.1 The next set of questions ask about your sexual behavior. Please answer these questions honestly to the best of your knowledge.   Remember, your name does not appear anywhere on this survey.

| Page Break |  |
| --- | --- |

Q11.2 How many male sexual partners have you had in the past 90 days?

________________________________________________________________

| Page Break |  |
| --- | --- |

Q11.3 How often do you use condoms?

- Never
- Sometimes
- About half the time
- Most of the time
- Always

| Page Break |  |
| --- | --- |

Q11.4 Have you had condomless receptive anal sex in the past 90 days?

- Yes
- No

| Page Break |  |
| --- | --- |

Q11.5 Have you ever been tested for HIV in your lifetime?

- Yes
- No

| Page Break |  |
| --- | --- |

Q11.6 About how long ago were you tested for HIV? 
Enter an approximate date in MM/DD/YYYY format (e.g., 07/01/2018). If you're unsure, provide your best guess.

________________________________________________________________

| Page Break |  |
| --- | --- |

Q11.7 If you have not been tested for HIV, which one of the following would you say is the MAIN reason why you have not been tested?

- It's unlikely you've been exposed to HIV
- You are afraid to find out if you were HIV positive
- You didn't want to think about HIV or about being HIV positive
- You were worried your name would be reported to the government if you tested positive
- You don't like needles
- You don't trust the results to be confidential
- You didn't know where to get tested
- Some other reason. Please specify ________________________________________________

| Page Break |  |
| --- | --- |

End of Block: Sexual Behavior & Risk Behavior

Start of Block: TAPS I

Q12.1 The next set of questions will ask you about alcohol and drug use.

| Page Break |  |
| --- | --- |

Q12.2 In the PAST 12 MONTHS, how often have you had 5 or more drinks containing alcohol in one day? 
***Note:*** *One standard drink is 1 small glass of wine (5 oz.), 1 beer (12 oz.), or 1 single shot of liquor.*

- Daily or Almost Daily
- Weekly
- Monthly
- Less than Monthly
- Never

| Page Break |  |
| --- | --- |

Q12.3 In the PAST 12 MONTHS, how often have you used any drugs including marijuana, cocaine or crack, heroin, methamphetamine (crystal meth), hallucinogens, ecstasy/MDMA?

- Daily or Almost Daily
- Weekly
- Monthly
- Less than Monthly
- Never

| Page Break |  |
| --- | --- |

Q12.4 In the PAST 12 MONTHS, how often have you used prescription medications for recreational use, more than was prescribed or that were not prescribed for you? 
Examples include: *Opiate pain relievers* (OxyContin, Vicodin, Percocet, Methadone) *Anxiety or sleeping medications* (Xanax, Ativan, Klonopin) and *medications for ADHD* (Adderall or Ritalin).

- Daily or Almost Daily
- Weekly
- Monthly
- Less than Monthly
- Never

End of Block: TAPS I

Start of Block: TAPS II

Q13.1 In the PAST 3 MONTHS, did you have a drink containing alcohol?

- Yes
- No

| Page Break |  |
| --- | --- |

Q13.2 In the PAST 3 MONTHS, did you have 5 or more drinks containing alcohol in a day?

- Yes
- No

| Page Break |  |
| --- | --- |

Q13.3 In the PAST 3 MONTHS, have you tried and failed to control, cut down or stop drinking?

- Yes
- No

| Page Break |  |
| --- | --- |

Q13.4 In the PAST 3 MONTHS, has anyone expressed concern about your drinking?

- Yes
- No

| Page Break |  |
| --- | --- |

Q13.5 In the PAST 3 MONTHS, did you use marijuana (hash, weed)?

- Yes
- No

| Page Break |  |
| --- | --- |

Q13.6 In the PAST 3 MONTHS, have you had a strong desire or urge to use marijuana at least once a week or more often?

- Yes
- No

| Page Break |  |
| --- | --- |

Q13.7 In the PAST 3 MONTHS, has anyone expressed concern about your use of marijuana?

- Yes
- No

| Page Break |  |
| --- | --- |

Q13.8 In the PAST 3 MONTHS, did you use cocaine, crack, or methamphetamine (crystal meth)?

- Yes
- No

| Page Break |  |
| --- | --- |

Q13.9 In the PAST 3 MONTHS, did you use cocaine, crack, or methamphetamine (crystal meth) at least once a week or more often?

- Yes
- No

| Page Break |  |
| --- | --- |

Q13.10 In the PAST 3 MONTHS, has anyone expressed concern about your use of cocaine, crack, or methamphetamine (crystal meth)?

- Yes
- No

| Page Break |  |
| --- | --- |

Q13.11 In the PAST 3 MONTHS, did you use heroin?

- Yes
- No

| Page Break |  |
| --- | --- |

Q13.12 In the PAST 3 MONTHS, have you tried and failed to control, cut down or stop using heroin?

- Yes
- No

| Page Break |  |
| --- | --- |

Q13.13 In the PAST 3 MONTHS, has anyone expressed concern about your use of heroin?

- Yes
- No

| Page Break |  |
| --- | --- |

Q13.14 In the PAST 3 MONTHS, did you use a prescription opiate pain reliever (for example, Percocet, Vicodin) not as prescribed or that was not prescribed for you?

- Yes
- No

| Page Break |  |
| --- | --- |

Q13.15 In the PAST 3 MONTHS, have you tried and failed to control, cut down or stop using an opiate pain reliever?

- Yes
- No

| Page Break |  |
| --- | --- |

Q13.16 In the PAST 3 MONTHS, has anyone expressed concern about your use of an opiate pain reliever?

- Yes
- No

| Page Break |  |
| --- | --- |

Q13.17 In the PAST 3 MONTHS, did you use a medication for anxiety or sleep (for example, Xanax, Ativan, or Klonopin) not as prescribed or that was not prescribed for you?

- Yes
- No

| Page Break |  |
| --- | --- |

Q13.18 In PAST 3 MONTHS, have you had a strong desire or urge to use medications for anxiety or sleep at least once a week or more often?

- Yes
- No

| Page Break |  |
| --- | --- |

Q13.19 In the PAST 3 MONTHS, has anyone expressed concern about your use of medication for anxiety or sleep?

- Yes
- No

| Page Break |  |
| --- | --- |

Q13.20 In the PAST 3 MONTHS, did you use a medication for ADHD (for example, Adderall, Ritalin) not as prescribed or that was not prescribed for you?

- Yes
- No

| Page Break |  |
| --- | --- |

Q13.21 In the PAST 3 MONTHS, did you use a medication for ADHD (for example, Adderall, Ritalin) at least once a week or more often?

- Yes
- No

| Page Break |  |
| --- | --- |

Q13.22 In the PAST 3 MONTHS, has anyone expressed concern about your use of a medication for ADHD (for example, Adderall or Ritalin)?

- Yes
- No

| Page Break |  |
| --- | --- |

Q13.23 In the PAST 3 MONTHS, did you use any other illegal or recreational drug (for example, ecstasy/molly, GHB, poppers, LSD, mushrooms, special K, bath salts, synthetic marijuana ('spice'), whip-its, etc.)?

- Yes
- No

| Page Break |  |
| --- | --- |

Q13.24 In the PAST 3 MONTHS, what were the other drug(s) you used?
Please specify:

________________________________________________________________

End of Block: TAPS II

Start of Block: Stigma

Q14.1 Tell us how you feel about the following statements.

| Page Break |  |
| --- | --- |

Q14.2 I feel afraid of people living with HIV/AIDS.

- Strongly Agree
- Agree
- Somewhat agree
- Neither agree nor disagree
- Somewhat disagree
- Disagree
- Strongly disagree

| Page Break |  |
| --- | --- |

Q14.3 I could not be friends with someone who has HIV/AIDS.

- Strongly agree
- Agree
- Somewhat agree
- Neither agree nor disagree
- Somewhat disagree
- Disagree
- Strongly disagree

| Page Break |  |
| --- | --- |

Q14.4 People who get HIV/AIDS through sex or drug use got what they deserve.

- Strongly agree
- Agree
- Somewhat agree
- Neither agree nor disagree
- Somewhat disagree
- Disagree
- Strongly disagree

| Page Break |  |
| --- | --- |

Q14.5 I feel anger toward people with HIV/AIDS.

- Strongly agree
- Agree
- Somewhat agree
- Neither agree nor disagree
- Somewhat disagree
- Disagree
- Strongly disagree

| Page Break |  |
| --- | --- |

End of Block: Stigma

Start of Block: Opinions on HIV testing

Q15.1 Which of these statements is most true for you?

- I don't see any need to regularly test for HIV
- I think I should get tested for HIV regularly, but I am not sure
- I'm ready to start getting regularly tested for HIV
- I'm trying to get tested regularly for HIV
- I've been getting tested for HIV regularly over the past few years

| Page Break |  |
| --- | --- |

Q15.2 Tell us if you "Agree" or "Disagree" with the following statements.

| Page Break |  |
| --- | --- |

Q15.3 Getting tested for HIV helps people feel better.

- Agree
- Disagree

| Page Break |  |
| --- | --- |

Q15.4 Getting tested for HIV helps people from getting HIV.

- Agree
- Disagree

| Page Break |  |
| --- | --- |

Q15.5 People in my life would leave if I had HIV.

- Agree
- Disagree

| Page Break |  |
| --- | --- |

Q15.6 People who tested positive for HIV should hide it from others.

- Agree
- Disagree

| Page Break |  |
| --- | --- |

Q15.7 I would rather not know if I have HIV.

- Agree
- Disagree

End of Block: Opinions on HIV testing

Start of Block: Attitudes towards HIV treatment

Q16.1
The following questions are about your opinions on HIV treatment. Please respond to each statement using a 7-point scale ranging from 1 “strongly disagree” to 7 “strongly agree.”
 
Since highly effective antiviral treatment for HIV (HAART)...

|  | strongly disagree | strongly agree |
| --- | --- | --- |

|  | 1 | 2 | 3 | 3 | 4 | 5 | 6 | 6 | 7 |
| --- | --- | --- | --- | --- | --- | --- | --- | --- | --- |

| I am less threatened by the idea of being HIV positive than I used to be. | 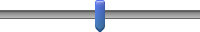 |
| --- | --- |
| I am less worried about HIV infection than I used to be | 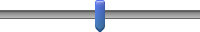 |
| I think HIV/AIDS is less of a problem than it used to be | 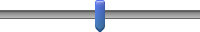 |
| I think HIV/AIDS is a less serious threat than it used to be because of new HIV/AIDS treatments | 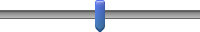 |
| I am much less concerned about becoming HIV positive myself because of new HIV/AIDS treatments | 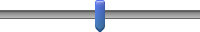 |
| I think that condom use during sex is less necessary now that new HIV/AIDS treatments are available | 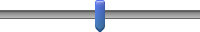 |
| I think that someone who is HIV positive now needs to care less about condom use | 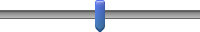 |
| I think that the need for condom use is less than it used to be, because you can always start new treatments | 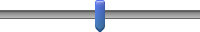 |
| I think that someone who is HIV positive and uses new HIV/AIDS treatments can be cured | 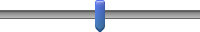 |
| I think that new HIV/AIDS treatments can eradicate the virus from your body | 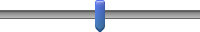 |

End of Block: Attitudes towards HIV treatment

Start of Block: Medical Mistrust

Q17.1 You’d better be cautious when dealing with health care organizations.

- Strongly agree
- Agree
- Disagree
- Strongly disagree

| Page Break |  |
| --- | --- |

Q17.2 Patients have sometimes been deceived or misled by health care organizations.

- Strongly agree
- Agree
- Disagree
- Strongly disagree

| Page Break |  |
| --- | --- |

Q17.3 When health care organizations make mistakes they usually cover it up.

- Strongly agree
- Agree
- Disagree
- Strongly disagree

| Page Break |  |
| --- | --- |

Q17.4 Health care organizations have sometimes done harmful experiments on patients without their knowledge.

- Strongly agree
- Agree
- Disagree
- Strongly disagree

| Page Break |  |
| --- | --- |

Q17.5 Health care organizations don’t always keep your information totally private.

- Strongly agree
- Agree
- Disagree
- Strongly disagree

| Page Break |  |
| --- | --- |

Q17.6 Sometimes I wonder if health care organizations really know what they are doing.

- Strongly agree
- Agree
- Disagree
- Strongly disagree

| Page Break |  |
| --- | --- |

Q17.7 Mistakes are common in health care organizations.

- Strongly agree
- Agree
- Disagree
- Strongly disagree

End of Block: Medical Mistrust

Start of Block: Sexual Delay Discounting

Q18.1
For this task, we will ask you questions about your willingness to have sex in various pretend situations.   

For the purpose of this task, please pretend that you are single and available, and that you are not cheating on anybody if you say you would have sex with somebody in this task.

| Page Break |  |
| --- | --- |

Q18.2 Think about how attractive each person below is. Based on just physical appearance, please think about whether each person is someone that you would have sex with if you liked the person’s personality and the time was right. 

Look at the pictures then click on the picture of the person you **MOST want to have sex with**.

- Image:9.jpg [Picture of a man]
- Image:53.jpg [Picture of a man]
- Image:52.jpg [Picture of a man]
- Image:58.jpg [Picture of a man]
- Image:45.jpg [Picture of a man]
- Image:27.jpg [Picture of a man]
- Image:59.jpg [Picture of a man]
- Image:20.jpg [Picture of a man]
- Image:35.jpg [Picture of a man]
- Image:60.jpg [Picture of a man]
- Image:51.jpg [Picture of a man]
- Image:28.jpg [Picture of a man]
- Image:22.jpg [Picture of a man]
- Image:15.jpg [Picture of a man]
- Image:41.jpg [Picture of a man]
- Image:26.jpg [Picture of a man]
- Image:17.jpg [Picture of a man]
- Image:43.jpg [Picture of a man]
- Image:50.jpg [Picture of a man]
- Image:56.jpg [Picture of a man]

| Page Break |  |
| --- | --- |

Q18.3
Imagine that a condom is available now. You can either have sex with this person **now** **without a condom** or you can have sex with this person **now** **with a condom**. Please click and drag in the bar below to rate how likely you are to have sex now without a condom versus having sex now with a condom.

 
This is the person you **most want to have sex with**.
[Picture of the man selected for question 17.2]

|  | I will definitely  have sex with  this person **now**  **without a condom**. | I will definitely  have sex with  this person **now**  **with a condom**. |
| --- | --- | --- |

|  | 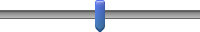 |
| --- | --- |

| Page Break |  |
| --- | --- |

Q18.4 Imagine that a condom is **NOT** available now. You can either have sex with this person **now** without a condom. Or, you can wait until you see them again in **1 hour**when you will have a condom. Please rate how likely you are to have **sex now without a condom** versus **having sex in 1 hour with a condom**.
 
This is the person you **most want to have sex with.** [Picture of the man selected for question 17.2]

|  | I will definitely  have sex with  this person **now  without a condom**. | I will definitely  **wait 1 hour**  to have sex  with this person  **with a condom**. |
| --- | --- | --- |

|  | 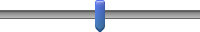 |
| --- | --- |

| Page Break |  |
| --- | --- |

Q18.5 Imagine that a condom is **NOT** available now. You can either have sex with this person **now** without a condom. Or, you can wait until you see them again in **3 hours**when you will have a condom. Please rate how likely you are to have **sex now without a condom** versus **having sex in 3 hours with a condom**.
 
This is the person you **most want to have sex with.**[Picture of the man selected for question 17.2]

|  | I will definitely  have sex with  this person **now  without a condom**. | I will definitely  **wait 3 hours**  to have sex  with this person  **with a condom**. |
| --- | --- | --- |

|  | 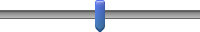 |
| --- | --- |

| Page Break |  |
| --- | --- |

Q18.6 Imagine that a condom is **NOT** available now. You can either have sex with this person **now** without a condom. Or, you can wait until you see them again in **6 hours**when you will have a condom. Please rate how likely you are to have **sex now without a condom** versus **having sex in 6 hours with a condom**.
 
This is the person you **most want to have sex with.**[Picture of the man selected for question 17.2]

|  | I will definitely  have sex with  this person **now  without a condom**. | I will definitely  **wait 6 hours**  to have sex  with this person  **with a condom**. |
| --- | --- | --- |

|  | 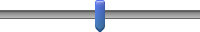 |
| --- | --- |

| Page Break |  |
| --- | --- |

Q18.7 Imagine that a condom is **NOT** available now. You can either have sex with this person **now** without a condom. Or, you can wait until you see them again in **1 day**when you will have a condom. Please rate how likely you are to have **sex now without a condom** versus **having sex in 1 day with a condom**.
 
This is the person you **most want to have sex with.**[Picture of the man selected for question 17.2]

|  | I will definitely  have sex with  this person **now  without a condom**. | I will definitely  **wait 1 day**  to have sex  with this person  **with a condom**. |
| --- | --- | --- |

|  | 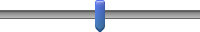 |
| --- | --- |

| Page Break |  |
| --- | --- |

Q18.8 Imagine that a condom is **NOT** available now. You can either have sex with this person **now** without a condom. Or, you can wait until you see them again in **1 week**when you will have a condom. Please rate how likely you are to have **sex now without a condom** versus **having sex in 1 week with a condom**.
 
This is the person you **most want to have sex with.**[Picture of the man selected for question 17.2]

|  | I will definitely  have sex with  this person **now  without a condom**. | I will definitely  **wait 1 week**  to have sex  with this person  **with a condom**. |
| --- | --- | --- |

|  | 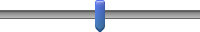 |
| --- | --- |

| Page Break |  |
| --- | --- |

Q18.9 Imagine that a condom is **NOT** available now. You can either have sex with this person **now** without a condom. Or, you can wait until you see them again in **1 month**when you will have a condom. Please rate how likely you are to have **sex now without a condom** versus **having sex in 1 month with a condom**.
 
This is the person you **most want to have sex with.**[Picture of the man selected for question 17.2]

|  | I will definitely  have sex with  this person **now  without a condom**. | I will definitely  **wait 1 month**  to have sex  with this person  **with a condom**. |
| --- | --- | --- |

|  | 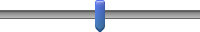 |
| --- | --- |

| Page Break |  |
| --- | --- |

Q18.10 Imagine that a condom is **NOT** available now. You can either have sex with this person **now** without a condom. Or, you can wait until you see them again in **3 months**when you will have a condom. Please rate how likely you are to have **sex now without a condom** versus **having sex in 3 months with a condom**.
 
This is the person you **most want to have sex with.**[Picture of the man selected for question 17.2]

|  | I will definitely  have sex with  this person **now  without a condom**. | I will definitely  **wait 3 months**  to have sex  with this person  **with a condom**. |
| --- | --- | --- |

|  | 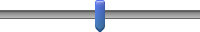 |
| --- | --- |

End of Block: Sexual Delay Discounting

14-Day Follow-Up

Start of Block: Welcome

Q1.1
Hello!

This is the first follow-up survey of the study.

Please answer the following questions. Your responses are confidential.

We will send you an email with your gift card within 72 hours after you complete this questionnaire.

Thank you!

End of Block: Welcome

Start of Block: Test kit use

Q2.1 Has your HIV self-test kit been used?

- Yes
- No

| Page Break |  |
| --- | --- |

|  |
| --- |

Q2.2 Who used the test kit? *[Shown if participant reports the HIV self-test kit has been used]*

- I used it.
- My partner used it.
- A friend used it.
- Other, please specify. ________________________________________________

| Page Break |  |
| --- | --- |

Q2.3 Why didn't you use your test kit? Select the one that is most true for you. *[Shown if participant reports NOT using the HIV self-test kit]*

- I already received testing somewhere else, such as a doctor's office or clinic.
- I changed my mind, and I don't want to take the test.
- I ordered my test kit, but I haven't received it yet.
- I haven't ordered my kit yet.
- Other, please explain. ________________________________________________

End of Block: Test kit use

Start of Block: Other testing

Q3.1 Where did you go to get testing? *[Shown if participant reports NOT using the HIV self-test kit because they tested somewhere else]*

- My doctor
- Community clinic
- Department of Public Health clinic
- Emergency department
- Telehealth provider, such as Plushcare
- Other ________________________________________________

| Page Break |  |
| --- | --- |

Q3.2 What was the result of this test? *[Shown if participant reports other testing]*

- Positive for HIV
- Negative for HIV
- Still waiting for results

End of Block: other testing

Start of Block: Rapid HIV Test Result

|  |
| --- |

Q4.1
What was the result of your HIV self-test? *[Shown if participant reported using the HIV self-test kit]*
 
Use the picture to help you remember or read your result.

- Positive
- Negative
- The test didn't work
- I couldn't understand the result

| Page Break |  |
| --- | --- |

Q4.2 Upload a photograph of your result here. *[Shown if participant reported using the HIV self-test kit]*

| Page Break |  |
| --- | --- |

Q4.3 When did you take the self-test? *[Shown if participant reported using the HIV self-test kit]*

If you're not sure, just give your best guess.

|  |
| --- |

Q4.4 Enter a date: *[Shown if participant reported using the HIV self-test kit]*

________________________________________________________________

End of Block: Rapid HIV Test Result

Start of Block: Confirmatory testing

Q5.1 Did you visit a doctor or clinic to get confirmatory tests for HIV? *[Shown if participant reports positive or indeterminate result]*

- Yes
- No

| Page Break |  |
| --- | --- |

Q5.2 Where did you go to get more tests? *[Shown if participant reports confirmatory testing]*

- My doctor
- Community clinic
- Department of Public Health clinic
- Emergency department
- Telehealth provider, such as Plushcare
- Other

| Page Break |  |
| --- | --- |

Q5.3 When did you go to get an HIV test from a doctor or clinic? *[Shown if participant reports confirmatory testing]*

If you're not sure, just give your best guess.

Q5.4 Enter a date:

________________________________________________________________

| Page Break |  |
| --- | --- |

Q5.5
What was the result of the HIV test from the doctor or clinic? *[Shown if participant reports confirmatory testing]*

- Positive for HIV
- Negative for HIV
- Waiting for results

| Page Break |  |
| --- | --- |

Q5.6 Did you start treatment for HIV infection? *[Shown if participant does NOT report a negative test results on confirmatory testing]*

- Yes
- No

| Page Break |  |
| --- | --- |

Q5.7 When did you start treatment for HIV? *[Shown if participant reports starting treatment]*

If you're not sure, just give your best guess.

Q5.8 Enter a date:

________________________________________________________________

End of Block: Confirmatory testing

Start of Block: PrEP use

Q6.1 Did you review the flyer on PrEP that came along with the test kit? *[Shown if participant does NOT report a positive test result]*

- Yes
- No

| Page Break |  |
| --- | --- |

Q6.2 Did you visit a clinic or doctor to discuss starting PrEP? *[Shown if participant does NOT report a positive test result]*

Select 'Yes' if you used the coupon code we sent you for PlushCare or used another telehealth provider (like Nurx).

- Yes
- No

| Page Break |  |
| --- | --- |

Q6.3 When did you see a doctor to discuss PrEP? *[Shown if participant reports visiting a provider about PrEP]*

If you're not sure, just give your best guess.

Q6.4 Enter a date:

________________________________________________________________

| Page Break |  |
| --- | --- |

Q6.5 Did you start taking PrEP? *[Shown if participant does NOT report a positive test result]*

- Yes
- No

| Page Break |  |
| --- | --- |

Q6.6 When did you start taking PrEP? *[Shown if participant reports starting PrEP]*

If you're not sure, just give your best guess.

Q6.7 Enter a date:

________________________________________________________________

End of Block: PrEP use

Start of Block: Opinions about PrEP

Q7.1 *[Shown if participant does NOT report a positive test result]*
The following statements express opinions about PrEP.

Let us know how much you agree or disagree with each statement.

| Page Break |  |
| --- | --- |

Q7.2 Taking a daily HIV pill is a good way to protect myself from getting HIV. *[Shown if participant does NOT report a positive test result]*

- Strongly agree
- Somewhat agree
- Neither agree nor disagree
- Somewhat disagree
- Strongly disagree

| Page Break |  |
| --- | --- |

Q7.3 By taking PrEP, I am lowering my chances of getting infected with HIV. *[Shown if participant does NOT report a positive test result]*

- Strongly agree
- Somewhat agree
- Neither agree nor disagree
- Somewhat disagree
- Strongly disagree

| Page Break |  |
| --- | --- |

Q7.4 Taking PrEP means I can have sex without using condoms. *[Shown if participant does NOT report a positive test result]*

- Strongly agree
- Somewhat agree
- Neither agree nor disagree
- Somewhat disagree
- Strongly disagree

| Page Break |  |
| --- | --- |

|  |
| --- |

Q7.5 Having PrEP available makes safer sex less important. *[Shown if participant does NOT report a positive test result]*

- Strongly agree
- Somewhat agree
- Neither agree nor disagree
- Somewhat disagree
- Strongly disagree

| Page Break |  |
| --- | --- |

Q7.6 Taking PrEP makes me more likely to have sex without using a condom. *[Shown if participant does NOT report a positive test result]*

- Strongly agree
- Somewhat agree
- Neither agree nor disagree
- Somewhat disagree
- Strongly disagree

| Page Break |  |
| --- | --- |

Q7.7 I feel uncomfortable taking HIV medication when I don’t have HIV. *[Shown if participant does NOT report a positive test result]*

- Strongly agree
- Somewhat agree
- Neither agree nor disagree
- Somewhat disagree
- Strongly disagree

| Page Break |  |
| --- | --- |

Q7.8 I worry that others will think I have HIV if they find out I am on PrEP. *[Shown if participant does NOT report a positive test result]*

- Strongly agree
- Somewhat agree
- Neither agree nor disagree
- Somewhat disagree
- Strongly disagree

| Page Break |  |
| --- | --- |

Q7.9 I worry about what other people would think of me if they knew I was on PrEP. *[Shown if participant does NOT report a positive test result]*

- Strongly agree
- Somewhat agree
- Neither agree nor disagree
- Somewhat disagree
- Strongly disagree

| Page Break |  |
| --- | --- |

Q7.10 I am ashamed to tell others that I am on PrEP. *[Shown if participant does NOT report a positive test result]*

- Strongly agree
- Somewhat agree
- Neither agree nor disagree
- Somewhat disagree
- Strongly disagree

| Page Break |  |
| --- | --- |

Q7.11 Not knowing if there are long-term side effects of taking a daily HIV pill makes me very uncomfortable. *[Shown if participant does NOT report a positive test result]*

- Strongly agree
- Somewhat agree
- Neither agree nor disagree
- Somewhat disagree
- Strongly disagree

| Page Break |  |
| --- | --- |

Q7.12 I would be more comfortable using PrEP if I just knew how it would affect my health. *[Shown if participant does NOT report a positive test result]*

- Strongly agree
- Somewhat agree
- Neither agree nor disagree
- Somewhat disagree
- Strongly disagree

| Page Break |  |
| --- | --- |

Q7.13 PrEP is too expensive. *[Shown if participant does NOT report a positive test result]*

- Strongly agree
- Somewhat agree
- Neither agree nor disagree
- Somewhat disagree
- Strongly disagree

| Page Break |  |
| --- | --- |

Q7.14 Gay and bisexual men who take PrEP are being responsible. *[Shown if participant does NOT report a positive test result]*

- Strongly agree
- Somewhat agree
- Neither agree nor disagree
- Somewhat disagree
- Strongly disagree

| Page Break |  |
| --- | --- |

Q7.15 PrEP is an excuse for gay and bisexual men to avoid using condoms. *[Shown if participant does NOT report a positive test result]*

- Strongly agree
- Somewhat agree
- Neither agree nor disagree
- Somewhat disagree
- Strongly disagree

| Page Break |  |
| --- | --- |

Q7.16 Gay and bisexual men who take PrEP are risk-takers. *[Shown if participant does NOT report a positive test result]*

- Strongly agree
- Somewhat agree
- Neither agree nor disagree
- Somewhat disagree
- Strongly disagree

| Page Break |  |
| --- | --- |

Q7.17 PrEP gives gay and bisexual men more options to remain safe. *[Shown if participant does NOT report a positive test result]*

- Strongly agree
- Somewhat agree
- Neither agree nor disagree
- Somewhat disagree
- Strongly disagree

| Page Break |  |
| --- | --- |

Q7.18 Gay and bisexual men who take PrEP are promiscuous. *[Shown if participant does NOT report a positive test result]*

- Strongly agree
- Somewhat agree
- Neither agree nor disagree
- Somewhat disagree
- Strongly disagree

| Page Break |  |
| --- | --- |

Q7.19 PrEP is only partially effective. *[Shown if participant does NOT report a positive test result]*

- Strongly agree
- Somewhat agree
- Neither agree nor disagree
- Somewhat disagree
- Strongly disagree

End of Block: Opinions about PrEP

Start of Block: PrEP Barriers and Facilitators

Q8.1 The following is a list of concerns people have about taking PrEP. Please read each item and rate the extent to which each concern is important **to you**. *[Shown if participant does NOT report a positive test result]*

|  | Not at all important | Slightly important | Moderately important | Very important | Extremely important |
| --- | --- | --- | --- | --- | --- |
| Having to take a pill every day |  |  |  |  |  |
| Potentially experiencing side effects |  |  |  |  |  |
| PrEP not protecting me against HIV infection completely |  |  |  |  |  |
| PrEP might make me more willing to have anal sex without a condom |  |  |  |  |  |
| Taking PrEP might make my partner(s) expect me to have anal sex without a condom |  |  |  |  |  |
| Having to take PrEP means I’m putting myself at risk for HIV |  |  |  |  |  |
| People will see me taking the medication and will want to know why I’m taking it |  |  |  |  |  |
| People will see me taking the medication and think that I have HIV |  |  |  |  |  |
| I don’t want to talk to my doctor about my sex life |  |  |  |  |  |
| Potentially having long-term health effects because of PrEP |  |  |  |  |  |
| If I do become HIV+, certain medicines won’t work because I was taking PrEP |  |  |  |  |  |
| I do not really think I am at high enough risk for HIV to warrant taking PrEP |  |  |  |  |  |
| I would be ashamed to take PrEP |  |  |  |  |  |

| Page Break |  |
| --- | --- |

Q8.2 The following is a list of things that might have made it easier for you to decide to take PrEP.
Please read each item and rate it according to whether it played a role in your decision to start or continue taking PrEP. *[Shown if participant does NOT report a positive test result]*

|  | Extremely important | Very important | Moderately important | Slightly important | Not at all important |
| --- | --- | --- | --- | --- | --- |
| Not having to pay for PrEP or because it is affordable |  |  |  |  |  |
| Getting free HIV/STD testing while I am on PrEP |  |  |  |  |  |
| Getting free one-on-one counseling and support while I am on PrEP |  |  |  |  |  |
| Not having to go to my regular doctor to get PrEP |  |  |  |  |  |
| Hearing from my doctor that taking PrEP would be best for my health |  |  |  |  |  |
| I am concerned that, even if I use condoms, I could still get HIV |  |  |  |  |  |
| I am a gay/bisexual guy or a transwoman and I know I am at really high risk for HIV |  |  |  |  |  |
| Hearing from my friends that they had good experiences with PrEP |  |  |  |  |  |

End of Block: PrEP Barriers and Facilitators

Start of Block: Refer for confirmatory testing

Q9.1 Confirming your home test result is very important. *[Shown if participant reports a positive or indeterminate result and does NOT report confirmatory testing]*

 It is important that you visit a clinic and do a blood test. This test will tell if you are infected with HIV or if the home test is wrong. If you need assistance, you may visit <https://gettested.cdc.gov/> to find a test site near you.

End of Block: Refer for confirmatory testing

Start of Block: Refer- Kit not used

Q10.1 Keeping yourself safe and healthy is important*.  [Shown if participant does NOT report using the HIV self-test kit]*
 
The HIV self-test or a test from a doctor or clinic will tell you if you have HIV so you can take action to keep yourself and your partners safe and healthy. 

| Page Break |  |
| --- | --- |

End of Block: Refer- Kit not used

Start of Block: End of survey

Q11.1 Thanks for answering questions for the first follow-up survey! We will send you a $25 gift card within 72 hours.

We'll contact you again in approximately six weeks for the final follow-up survey. You will receive another $25 gift card for completing the last survey.

Please click the arrow below to finish this survey.

End of Block: End of survey

60-Day Follow-Up*

*Subsets of questions in this survey are shown based on participants’ responses on the 14-day follow-up survey.

Start of Block: Welcome

Q1.1
Hello!

This is the last follow-up survey of the study.

Please answer the following questions. Your responses are confidential.

We will send you an email with your gift card within 72 hours after you complete this questionnaire.

Thank you!

End of Block: Welcome

Start of Block: Test kit use

Q2.1 Has your HIV self-test kit been used?

- Yes
- No

| Page Break |  |
| --- | --- |

|  |
| --- |

Q2.2 Who used the test kit? *[Shown if participant reports the HIV self-test kit has been used]*

- I used it.
- My partner used it.
- A friend used it.
- Other, please specify. ________________________________________________

| Page Break |  |
| --- | --- |

Q2.3 Why didn't you use your test kit? Select the one that is most true for you. *[Shown if participant reports NOT using the HIV self-test kit]*

- I already received testing somewhere else, such as a doctor's office or clinic.
- I changed my mind, and I don't want to take the test.
- I ordered my test kit, but I haven't received it yet.
- I haven't ordered my kit yet.
- Other, please explain. ________________________________________________

End of Block: Test kit use

Start of Block: Other testing

Q3.1 Where did you go to get testing? *[Shown if participant reports NOT using the HIV self-test kit because they tested somewhere else]*

- My doctor
- Community clinic
- Department of Public Health clinic
- Emergency department
- Telehealth provider, such as Plushcare
- Other ________________________________________________

| Page Break |  |
| --- | --- |

Q3.2 What was the result of this test? *[Shown if participant reports other testing]*

- Positive for HIV
- Negative for HIV
- Still waiting for results

End of Block: other testing

Start of Block: Rapid HIV Test Result

|  |
| --- |

Q4.1
What was the result of your HIV self-test? *[Shown if participant reported using the HIV self-test kit]*
 
Use the picture to help you remember or read your result.

- Positive
- Negative
- The test didn't work
- I couldn't understand the result

| Page Break |  |
| --- | --- |

Q4.2 Upload a photograph of your result here. *[Shown if participant reported using the HIV self-test kit]*

| Page Break |  |
| --- | --- |

Q4.3 When did you take the self-test? *[Shown if participant reported using the HIV self-test kit]*

If you're not sure, just give your best guess.

|  |
| --- |

Q4.4 Enter a date: *[Shown if participant reported using the HIV self-test kit]*

________________________________________________________________

End of Block: Rapid HIV Test Result

Start of Block: Confirmatory testing

Q5.1 Did you visit a doctor or clinic to get confirmatory tests for HIV? *[Shown if participant reports positive or indeterminate result]*

- Yes
- No

| Page Break |  |
| --- | --- |

Q5.2 Where did you go to get more tests? *[Shown if participant reports confirmatory testing]*

- My doctor
- Community clinic
- Department of Public Health clinic
- Emergency department
- Telehealth provider, such as Plushcare
- Other

| Page Break |  |
| --- | --- |

Q5.3 When did you go to get an HIV test from a doctor or clinic? *[Shown if participant reports confirmatory testing]*

If you're not sure, just give your best guess.

Q5.4 Enter a date:

________________________________________________________________

| Page Break |  |
| --- | --- |

Q5.5
What was the result of the HIV test from the doctor or clinic? *[Shown if participant reports confirmatory testing]*

- Positive for HIV
- Negative for HIV
- Waiting for results

| Page Break |  |
| --- | --- |

Q5.6 Did you start treatment for HIV infection? *[Shown if participant does NOT report a negative test results on confirmatory testing]*

- Yes
- No

| Page Break |  |
| --- | --- |

Q5.7 When did you start treatment for HIV? *[Shown if participant reports starting treatment]*

If you're not sure, just give your best guess.

Q5.8 Enter a date:

________________________________________________________________

End of Block: Confirmatory testing

Start of Block: PrEP use

Q6.1 Did you review the flyer on PrEP that came along with the test kit? *[Shown if participant does NOT report a positive test result]*

- Yes
- No

| Page Break |  |
| --- | --- |

Q6.2 Did you visit a clinic or doctor to discuss starting PrEP? *[Shown if participant does NOT report a positive test result]*

Select 'Yes' if you used the coupon code we sent you for PlushCare or used another telehealth provider (like Nurx).

- Yes
- No

| Page Break |  |
| --- | --- |

Q6.3 When did you see a doctor to discuss PrEP? *[Shown if participant reports visiting a provider about PrEP]*

If you're not sure, just give your best guess.

Q6.4 Enter a date:

________________________________________________________________

| Page Break |  |
| --- | --- |

Q6.5 Did you start taking PrEP? *[Shown if participant does NOT report a positive test result]*

- Yes
- No

| Page Break |  |
| --- | --- |

Q6.6 When did you start taking PrEP? *[Shown if participant reports starting PrEP]*

If you're not sure, just give your best guess.

Q6.7 Enter a date:

________________________________________________________________

End of Block: PrEP use

Start of Block: Opinions about PrEP

Q7.1 *[Shown if participant does NOT report a positive test result]*
The following statements express opinions about PrEP.

Let us know how much you agree or disagree with each statement.

| Page Break |  |
| --- | --- |

Q7.2 Taking a daily HIV pill is a good way to protect myself from getting HIV. *[Shown if participant does NOT report a positive test result]*

- Strongly agree
- Somewhat agree
- Neither agree nor disagree
- Somewhat disagree
- Strongly disagree

| Page Break |  |
| --- | --- |

Q7.3 By taking PrEP, I am lowering my chances of getting infected with HIV. *[Shown if participant does NOT report a positive test result]*

- Strongly agree
- Somewhat agree
- Neither agree nor disagree
- Somewhat disagree
- Strongly disagree

| Page Break |  |
| --- | --- |

Q7.4 Taking PrEP means I can have sex without using condoms. *[Shown if participant does NOT report a positive test result]*

- Strongly agree
- Somewhat agree
- Neither agree nor disagree
- Somewhat disagree
- Strongly disagree

| Page Break |  |
| --- | --- |

|  |
| --- |

Q7.5 Having PrEP available makes safer sex less important. *[Shown if participant does NOT report a positive test result]*

- Strongly agree
- Somewhat agree
- Neither agree nor disagree
- Somewhat disagree
- Strongly disagree

| Page Break |  |
| --- | --- |

Q7.6 Taking PrEP makes me more likely to have sex without using a condom. *[Shown if participant does NOT report a positive test result]*

- Strongly agree
- Somewhat agree
- Neither agree nor disagree
- Somewhat disagree
- Strongly disagree

| Page Break |  |
| --- | --- |

Q7.7 I feel uncomfortable taking HIV medication when I don’t have HIV. *[Shown if participant does NOT report a positive test result]*

- Strongly agree
- Somewhat agree
- Neither agree nor disagree
- Somewhat disagree
- Strongly disagree

| Page Break |  |
| --- | --- |

Q7.8 I worry that others will think I have HIV if they find out I am on PrEP. *[Shown if participant does NOT report a positive test result]*

- Strongly agree
- Somewhat agree
- Neither agree nor disagree
- Somewhat disagree
- Strongly disagree

| Page Break |  |
| --- | --- |

Q7.9 I worry about what other people would think of me if they knew I was on PrEP. *[Shown if participant does NOT report a positive test result]*

- Strongly agree
- Somewhat agree
- Neither agree nor disagree
- Somewhat disagree
- Strongly disagree

| Page Break |  |
| --- | --- |

Q7.10 I am ashamed to tell others that I am on PrEP. *[Shown if participant does NOT report a positive test result]*

- Strongly agree
- Somewhat agree
- Neither agree nor disagree
- Somewhat disagree
- Strongly disagree

| Page Break |  |
| --- | --- |

Q7.11 Not knowing if there are long-term side effects of taking a daily HIV pill makes me very uncomfortable. *[Shown if participant does NOT report a positive test result]*

- Strongly agree
- Somewhat agree
- Neither agree nor disagree
- Somewhat disagree
- Strongly disagree

| Page Break |  |
| --- | --- |

Q7.12 I would be more comfortable using PrEP if I just knew how it would affect my health. *[Shown if participant does NOT report a positive test result]*

- Strongly agree
- Somewhat agree
- Neither agree nor disagree
- Somewhat disagree
- Strongly disagree

| Page Break |  |
| --- | --- |

Q7.13 PrEP is too expensive. *[Shown if participant does NOT report a positive test result]*

- Strongly agree
- Somewhat agree
- Neither agree nor disagree
- Somewhat disagree
- Strongly disagree

| Page Break |  |
| --- | --- |

Q7.14 Gay and bisexual men who take PrEP are being responsible. *[Shown if participant does NOT report a positive test result]*

- Strongly agree
- Somewhat agree
- Neither agree nor disagree
- Somewhat disagree
- Strongly disagree

| Page Break |  |
| --- | --- |

Q7.15 PrEP is an excuse for gay and bisexual men to avoid using condoms. *[Shown if participant does NOT report a positive test result]*

- Strongly agree
- Somewhat agree
- Neither agree nor disagree
- Somewhat disagree
- Strongly disagree

| Page Break |  |
| --- | --- |

Q7.16 Gay and bisexual men who take PrEP are risk-takers. *[Shown if participant does NOT report a positive test result]*

- Strongly agree
- Somewhat agree
- Neither agree nor disagree
- Somewhat disagree
- Strongly disagree

| Page Break |  |
| --- | --- |

Q7.17 PrEP gives gay and bisexual men more options to remain safe. *[Shown if participant does NOT report a positive test result]*

- Strongly agree
- Somewhat agree
- Neither agree nor disagree
- Somewhat disagree
- Strongly disagree

| Page Break |  |
| --- | --- |

Q7.18 Gay and bisexual men who take PrEP are promiscuous. *[Shown if participant does NOT report a positive test result]*

- Strongly agree
- Somewhat agree
- Neither agree nor disagree
- Somewhat disagree
- Strongly disagree

| Page Break |  |
| --- | --- |

Q7.19 PrEP is only partially effective. *[Shown if participant does NOT report a positive test result]*

- Strongly agree
- Somewhat agree
- Neither agree nor disagree
- Somewhat disagree
- Strongly disagree

End of Block: Opinions about PrEP

Start of Block: PrEP Barriers and Facilitators

Q8.1 The following is a list of concerns people have about taking PrEP. Please read each item and rate the extent to which each concern is important **to you**. *[Shown if participant does NOT report a positive test result]*

|  | Not at all important | Slightly important | Moderately important | Very important | Extremely important |
| --- | --- | --- | --- | --- | --- |
| Having to take a pill every day |  |  |  |  |  |
| Potentially experiencing side effects |  |  |  |  |  |
| PrEP not protecting me against HIV infection completely |  |  |  |  |  |
| PrEP might make me more willing to have anal sex without a condom |  |  |  |  |  |
| Taking PrEP might make my partner(s) expect me to have anal sex without a condom |  |  |  |  |  |
| Having to take PrEP means I’m putting myself at risk for HIV |  |  |  |  |  |
| People will see me taking the medication and will want to know why I’m taking it |  |  |  |  |  |
| People will see me taking the medication and think that I have HIV |  |  |  |  |  |
| I don’t want to talk to my doctor about my sex life |  |  |  |  |  |
| Potentially having long-term health effects because of PrEP |  |  |  |  |  |
| If I do become HIV+, certain medicines won’t work because I was taking PrEP |  |  |  |  |  |
| I do not really think I am at high enough risk for HIV to warrant taking PrEP |  |  |  |  |  |
| I would be ashamed to take PrEP |  |  |  |  |  |

| Page Break |  |
| --- | --- |

Q8.2 The following is a list of things that might have made it easier for you to decide to take PrEP.
Please read each item and rate it according to whether it played a role in your decision to start or continue taking PrEP. *[Shown if participant does NOT report a positive test result]*

|  | Extremely important | Very important | Moderately important | Slightly important | Not at all important |
| --- | --- | --- | --- | --- | --- |
| Not having to pay for PrEP or because it is affordable |  |  |  |  |  |
| Getting free HIV/STD testing while I am on PrEP |  |  |  |  |  |
| Getting free one-on-one counseling and support while I am on PrEP |  |  |  |  |  |
| Not having to go to my regular doctor to get PrEP |  |  |  |  |  |
| Hearing from my doctor that taking PrEP would be best for my health |  |  |  |  |  |
| I am concerned that, even if I use condoms, I could still get HIV |  |  |  |  |  |
| I am a gay/bisexual guy or a transwoman and I know I am at really high risk for HIV |  |  |  |  |  |
| Hearing from my friends that they had good experiences with PrEP |  |  |  |  |  |

End of Block: PrEP Barriers and Facilitators

Start of Block: Social Media PrEP Evaluation Questions

| Page Break |  |
| --- | --- |

Q9.1 We would like to ask your opinions about your participation in this study.

| Page Break |  |
| --- | --- |

Q9.2
We offered study participants a free HIV self-test kit.
 
Had you ever used a self-test kit to test for HIV before this study?

- Yes
- No

| Page Break |  |
| --- | --- |

Q9.3 Other than the time you tested for this study, how many times in your life did you use an HIV home self-test kit?

- 1 time
- 2 times
- 3 times or more

Q9.4 Where did you get the HIV self-test kit?

- Pharmacy, such as Walgreens, CVS, etc.
- eBay
- Amazon
- Other, please describe ________________________________________________

| Page Break |  |
| --- | --- |

Q9.5 Had you ever heard about a home HIV self-test kit before this study?

- Yes
- No

| Page Break |  |
| --- | --- |

Q9.6 Was there a reason you hadn't gotten a home HIV test kit before joining this study?

- Hadn't considered getting a home test kit
- I don't know where to get one
- I think it's expensive
- I don't trust the home test kit result
- I prefer going to a clinic
- Other ________________________________________________

| Page Break |  |
| --- | --- |

Q9.7 Did you have any problems receiving the test kit?

- Yes
- No

| Page Break |  |
| --- | --- |

Q9.8
When you tested using the kit we sent you, how easy or difficult was using the HIV test kit?

- Extremely easy
- Somewhat easy
- Neither easy nor difficult
- Somewhat difficult
- Extremely difficult

| Page Break |  |
| --- | --- |

Q9.9 How likely are you to use a home HIV test kit in the future?

- Extremely likely
- Somewhat likely
- Neither likely nor unlikely
- Somewhat unlikely
- Extremely unlikely

| Page Break |  |
| --- | --- |

Q9.10 In this study, you were given the chance to order a test kit online and get PrEP online. How would you rate your experience?

- Extremely positive
- Somewhat positive
- Neither positive nor negative
- Somewhat negative
- Extremely negative

| Page Break |  |
| --- | --- |

Q9.11
We put advertisements in different places online to find study participants. In your opinion, what is the best way to reach men to provide information on HIV prevention or PrEP?

Please click and drag to rank the different sites. Make number 1 the best place to provide HIV or PrEP information. 

______ Facebook

______ Twitter

______ Instagram

______ Snapchat

______ Other social media sites

______ Grindr

______ Jack'd

______ Scruff

______ Other dating/hook up apps

______ Google

______ Bing

______ Yahoo

______ Other search engines

| Page Break |  |
| --- | --- |

Q9.12 Did you use your coupon to get an appointment with a PrEP provider?

- Yes
- No

| Page Break |  |
| --- | --- |

Q9.13 How soon do you think you will use your coupon?

- In the next 7 days
- The next 8 days up to the end of this month
- Some time between the next month and the following 6 months
- After 6 months
- I am not planning on using my coupon for PrEP

| Page Break |  |
| --- | --- |

Q9.14 How would you rate your experience making an online appointment with a provider to discuss PrEP?

- Extremely positive
- Somewhat positive
- Neither positive nor negative
- Somewhat negative
- Extremely negative

End of Block: Social Media PrEP Evaluation Questions

Start of Block: Refer for confirmatory testing

Q10.1 Confirming your home test result is very important. *[Shown if participant reports a positive or indeterminate result and does NOT report confirmatory testing]*

 It is important that you visit a clinic and do a blood test. This test will tell if you are infected with HIV or if the home test is wrong. If you need assistance, you may visit https://gettested.cdc.gov/ to find a test site near you.

End of Block: Refer for confirmatory testing

Start of Block: Refer- Kit not used

Q11.1 Keeping yourself safe and healthy is important*.  [Shown if participant does NOT report using the HIV self-test kit]*
 
The HIV self-test or a test from a doctor or clinic will tell you if you have HIV so you can take action to keep yourself and your partners safe and healthy. 

| Page Break |  |
| --- | --- |

End of Block: Refer- Kit not used

Start of Block: End of survey

Q12.1 Thanks for answering questions for the last follow-up survey! We will send you a $25 gift card within 72 hours.

Please click the arrow below to finish this survey.

End of Block: End of survey
